# Strategies to reduce the energy content of foods pre-ordered for lunch in the workplace: a randomised controlled trial in an experimental online canteen

**DOI:** 10.1186/s12966-022-01257-5

**Published:** 2022-05-12

**Authors:** Sarah Breathnach, Phillippa Lally, Clare H. Llewellyn, Alex Sutherland, Dimitrios A. Koutoukidis

**Affiliations:** 1 Research Department of Behavioural Science and Health, Faculty of Epidemiology & Public Health, University College London, 1-19 Torrington Place, London, WC1E 7HB UK; 2 Behavioural Insights Team, 4 Matthew Parker Street, SW1H 9NP London, UK; 3 Nuffield Department of Primary Care Health Sciences, University of Oxford NIHR Oxford Biomedical Research Centre, Oxford University Hospitals NHS Foundation, Oxford Trust, UK

**Keywords:** Swaps, Lower-energy, Canteen, Randomised controlled trial, PACE, Canteen, Workplace, Pre-ordering, Lunch

## Abstract

**Background:**

Prompting employees to swap their usual lunches for lower-energy alternatives may help align energy intake with public health recommendations. We tested the effect of offering lower-energy swaps with and without physical activity calorie equivalent (PACE) information on the energy of lunches pre-ordered in an online hypothetical workplace canteen.

**Methods:**

UK employed adults (*n* = 2,150) were invited to hypothetically pre-order their lunch from the canteen through a custom-made online platform. They were randomised 1:1:1 to: (i) control: no swaps offered; (ii) lower-energy swaps offered; or (iii) lower-energy swaps offered with PACE information. The primary outcome was the total energy ordered using analysis of covariance and controlling for the energy content of the initial items ordered. Secondary outcomes were swap acceptance rate and intervention acceptability.

**Results:**

Participants were 54% female, had a mean age of 36.8 (*SD* = 11.6) and a BMI of 26.3 (*SD* = 5.6). Compared with an average 819 kcal energy ordered in the control, both the swaps and swaps + PACE interventions significantly reduced average energy ordered by 47 kcal (95% CI: -82 to -13, *p* = 0.003) and 66 kcal (95% CI: -100 to -31, *p* < 0.001), respectively. Compared with offering swaps only, the swaps + PACE intervention led to significantly higher swap acceptance (OR: 1.63, 95% CI: 1.27 to 2.09, *p* < 0.001) but did not significantly reduce energy ordered (-19 kcal, 95% CI: -53 to 16, *p* = 0.591). About 65% and 16% of intervention participants found the swap interventions acceptable and unacceptable, respectively, with the swaps + PACE intervention being considered more acceptable than swaps only (OR: 1.32, 95%CI: 1.09 to 1.60, *p* < 0.004).

**Conclusion:**

Offering lower-energy swaps with or without PACE information reduced the energy of pre-ordered lunches experimentally. Both interventions hold promise for reducing the energy of purchased foods and drinks.

**Trial Registration**

As Predicted reference number: 56358, 22/01/21, https://aspredicted.org/pw2qr.pdf

**Supplementary Information:**

The online version contains supplementary material available at 10.1186/s12966-022-01257-5.

## Background

Excess weight increases morbidity and mortality [1]. Biological, behavioural, societal, and environmental factors interact leading to positive energy balance and excess weight. Strategies to reduce population energy intake may substantially contribute to halting the rise in obesity [2] Prompting consumers to swap their initial food and drink selections for lower-energy alternatives while shopping may help bring energy intake into line with public health recommendations. Swap-based interventions have been tested in experimental settings and the results show reductions in both the saturated fat [3] and salt [4] content of grocery baskets. More recently, offering swaps was shown to reduce the energy content of snacks and drinks ordered in an experimental online canteen [5]. This study also found that accompanying swaps offered with physical activity calorie equivalent (PACE) information, indicating the amount of energy contained in a food or drink and the amount of physical activity that would be required for it to be expended (i.e., “*How about a swap? Save [x] calories* = *[y] min walk”)*, significantly increased the likelihood that a swap offered would be accepted when compared to offering swaps with no specific information (i.e., participants were simply asked: “*How about a swap?*”). The provision of PACE information also increased intervention acceptability ratings. These findings indicate that providing easily interpretable or tangible information when offering lower-energy swaps for snacks or drinks increases their acceptance. Similar to online supermarkets, pre-ordering websites for canteens provide a platform for the delivery of health promotion interventions. Pre-ordering might become increasingly popular [6], particularly after the first waves of COVID-19, where companies may consider implementing pre-ordering systems to reduce the physical contact associated with long lunch queues at peak hours. However, little is known about (a) whether lower-energy swaps offered across a full canteen menu, including items such as hot meals or sandwiches, would be accepted; and (b), if swaps are accepted, whether consumers immediately compensate for energy reductions (e.g., by ordering more items and thus more energy) across their whole meal. Although field trials are considered the gold standard method of investigation, they are costly and challenging to conduct. Given the lack of research on swap-based interventions in canteen settings, we decided to use a field-lab hybrid study to perform an initial investigation of the potential effectiveness of the interventions which could be used to inform a future field trial.

A field-lab hybrid study is used to answer these research questions. Field-lab hybrid studies are hypothetical choice experiments usually delivered via online platforms that mimic real-world plausible scenarios. While they are not as tightly controlled as traditional lab experiments, nor do they test ‘real’ choices like field trials, they permit the testing of variables that would be difficult to examine in a field trial due to the pragmatic constraints that real-world settings inevitably impose. Field-lab hybrid studies allow for data to be easily collected from large numbers of participants, not only on the target behaviour but on key demographics. These studies provide estimates of potential intervention efficacy while also enabling researchers to ensure that proposed interventions do not enhance health inequalities or drive unexpected behaviours, evidence which is essential in helping to recruit field trial sites. Field-lab hybrid studies can demonstrate to potential sites that interventions are acceptable in principle to their customers, and are unlikely to damage profit margins while still benefiting the health of their customers. As such, field-lab hybrid studies represent an important middle ground of study that neither lab nor field studies can provide.

The aim of this study was to test the effect of (i) offering lower-energy swaps, and (ii) offering lower-energy swaps with a PACE message on the total energy of items pre-ordered for lunch within the context of an experimental online workplace canteen. We hypothesized that the Swap + PACE intervention would be more effective than offering swaps alone.

## Methods

### Design & setting

This pre-registered (AsPredicted ref: 56358, https://aspredicted.org/pw2qr.pdf), three-arm, randomised controlled trial was conducted in an experimental online canteen developed using REDCap (Research Electronic Data Capture), a web application for data collection [7]. The website was designed to simulate an online pre-ordering system for a real-world workplace canteen. An online canteen pre-ordering system is a website which displays the canteen’s menu and allows employees to place their lunch order in the morning for collection later that day. Participants were able to hypothetically order their lunch from 6 menus containing a selection of main hot meals (*n* = 3), jacket potatoes (*n* = 10), soup & sandwiches (*n* = 15), sweet snacks (*n* = 18), savoury snacks (*n* = 20), and non-alcoholic drinks (*n* = 18) based on the menus of a real-world workplace canteen with whom we partnered (see Additional file 1, Appendix D for full menus). In the real-world canteen, main hot meal options (*n* = 3) change on a daily basis. Participants were randomly assigned to view and choose from the main hot meals for 1 of 5 different days to reflect this. The CONSORT checklist is available in Additional files 2, 3, and 4.

### Participants

In February 2021, participants were recruited through Prolific Academic, an online participant sourcing platform [8]. To be eligible for the study, participants had to be ≥ 18 years, a UK resident, speak English fluently, and be in full or part-time employment. Those following restricted diets, e.g., vegetarian or dairy-free, were ineligible, as this would affect the acceptability of swaps offered. Prolific Academic pre-screened participants on these criteria and sent invitations to eligible panel members. Potential participants could also access the study via a link published on the Prolific Academic dashboards of all eligible panel members. Participants could follow this link where they were able to confirm their eligibility, read the information sheet, and provide consent.

### Randomisation & blinding

Simple randomisation (1:1:1) was performed using Predictiv [9]. Participants were randomised to both a trial arm (1 of 3) and a menu (1 of 5), meaning that participants were evenly allocated to 1 of 15 groups. To do this, the platform allocated eligible participants a random integer between 1 and 15 representing the 15 conditions. To ensure balance, the algorithm ranked the conditions (1–15) based on the number of participants previously allocated to each and allocated the next participant to one of the 7 least used conditions. While investigators were not blinded to condition, they were not able to manipulate any study parameters following the initial study set up, as all study procedures were automated.

### Online ordering task

Following randomisation, participants were directed to REDCap where they were asked to indicate their current subjective feeling of hunger. Participants responded to the question “how hungry do you feel” using a slider scale anchored with the extremes of “not at all” (0) on the far left, and “extremely” (100) on the far right [10]. Participants were then asked to imagine they worked for a company that had a pre-ordering website for their canteen and to order their lunch for the day using the website. They were asked to make choices that were in keeping with what they would typically have for lunch during their working day. Lower-energy swaps were automatically offered for originally selected menu items, if a suitable alternative was available. Participants placed one order only and did not pay for this order.

### Swaps offered

**Fig. 1:**
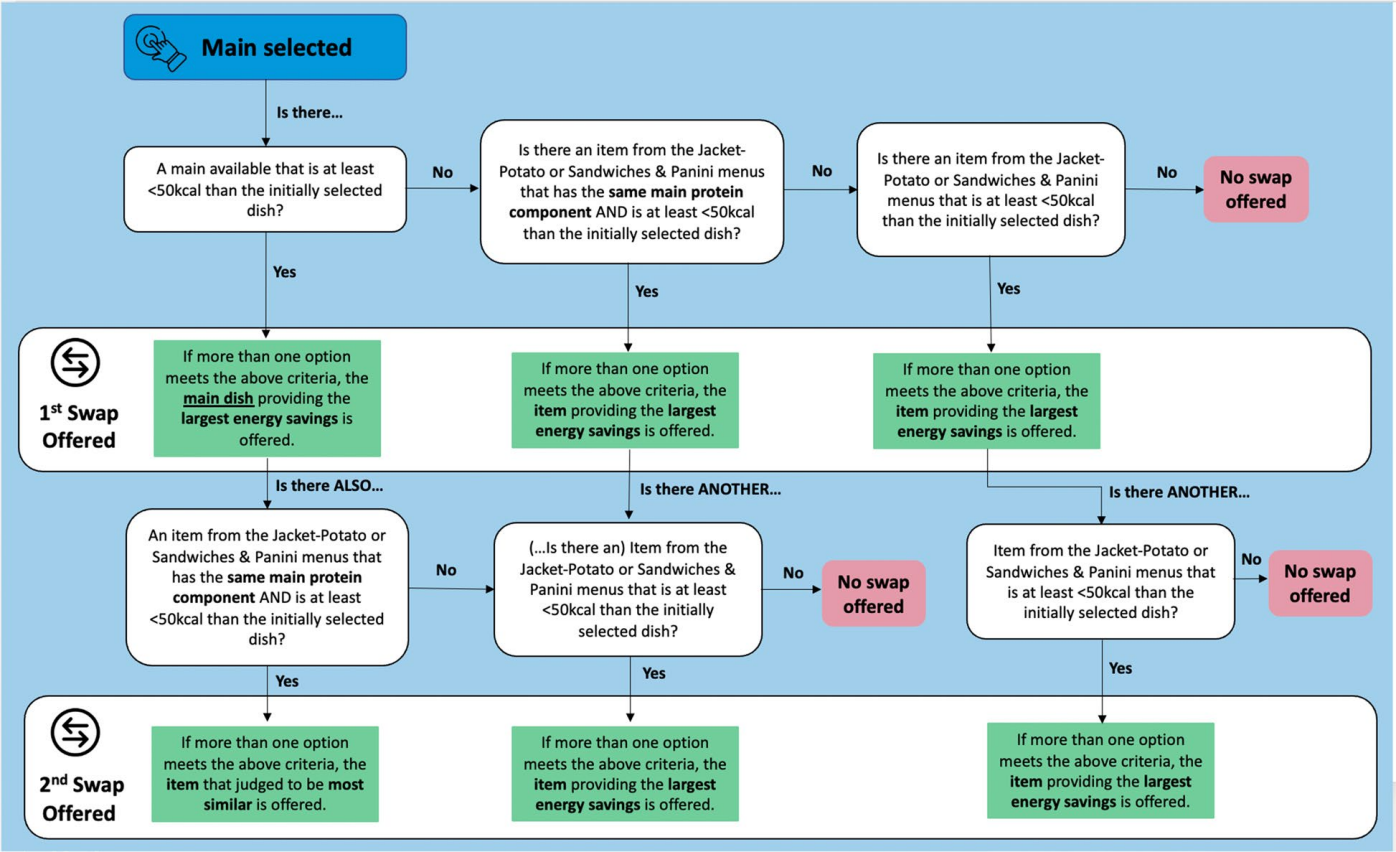
Flow diagram outlining the criteria for swaps offered for main dishes

Swaps offered were pre-determined by the research team using the criteria outlined in this section. The criteria for main hot meals differed to the ones in all other menus. Regardless of the menu, to qualify as a swap, the alternative had to contain at least 50 kcal less than the originally selected item, because a minimum of 50 kcal reduction per-person per-day has been identified as being clinically relevant [11]. Swaps offered were lower-energy items and were almost always from the same menu as the initial selection to ensure that the swap offered was as similar as possible to the initially selected item. Only one swap was offered for each item on a given menu, except for main hot meals where two swaps were offered. The criteria for each individual menu can be found in Sect. 6.2 of Additional file 1. Choices of swaps for main hot meals followed an algorithmic process as per Fig. 1. Main hot dishes served in the real-world canteen are dissimilar to each other to provide variety, therefore offering a swap from within the same menu results in offering a dissimilar meal. Two swaps were offered for each main dish to maximise the potential acceptance of swaps offered. In most cases, the first swap offered was the main with the lowest energy content and the second swap offered was either a jacket potato or an item from the soup and sandwich menu similar to the originally selected item. Similarity was based on the main protein source in the dish. Where possible, the main protein source was matched. For example, a chicken sandwich was offered as a swap for a chicken curry.

A registered dietitian analysed the energy content of menu items using recipes provided by our partner canteen’s catering company and the nutritional information published on the supplier’s website. A lower-energy swap was available for 71% of options (see Table 1). Swaps were offered immediately after selection. Participants could decline or accept the swap for all menus except the main hot menu by clicking either “*No, I will stick with my choice*” or “*Yes, I would like to swap*”. For the main hot menu, participants could decline or accept the swaps offered by clicking: “*No, I will stick with my choice*”, “*Yes, I would like to swap to [name of swap 1]” or* “*Yes, I would like to swap to [name of swap 2]”*.

**Table 1:** Menu categories with examples of swaps offered, % swaps available, means (range) of energy content

*Menu*	*Examples of items offered*	*Initially selected item*	*Swap(s) offered*	*% Swaps available*	*Mean (range) energy of menu items*
Main hot meals (*n* = 3)	1. Beef lasagne 2. Haddock with crushed potatoes, peas and broccoli 3.Moroccan style tomato & chickpea pie, mixed leaf salad	1.Beef lasagne, mixed leaf salad (680 kcal)	1.Haddock with crushed potatoes, peas and broccoli 415 kcal) 2. Beef, Horseradish & rocket sandwich (336 kcal)	100% (3/3)	595 kcal (415–831 kcal)
Jacket potatoes (*n* = 10)	1.Jacket potato with baked beans & cheese 2. Jacket potato with tuna mayo	1.Jacket potato with baked beans & cheese (633 kcal)	1. Jacket potato with baked beans (384 kcal)	80% (8/10)	478 kcal (275–728 kcal)
Soup & sandwiches (*n* = 15)	1. Bacon Lettuce Tomato sandwich 2. Chicken & stuffing sandwich 3.Tomato and basil soup	1.Bacon Lettuce Tomato sandwich (355 kcal)	1. Smoked ham & mustard sandwich (262 kcal)	80% (12/15)	350 kcal (235- 449 kcal)
Sweet snacks (*n* = 18)	1. Chocolate brownie 2.Yoghurt 3.Fruit salad	1.Chocolate brownie (283 kcal)	1.Broderick’s Chocolatey Solid Brick (217 kcal)	72% (13/18)	215 kcal (74- 471 kcal)
Savoury snacks (*n* = 20)	1.McCoys crisps 2.Eat real chips 3.Propercorn Popcorn	1.McCoys Flame Grilled Steak (252 kcal)	1.Popchips BBQ (97 kcal)	75% (15/20)	173 kcal (87- 261 kcal)
Drinks (*n* = 18)	1.Oasis Summer Fruits 2.Coca-cola 3.Water	1. Oasis Summer Fruits (86 kcal)	1. Oasis Summer Fruits Zero (17 kcal)	50% (9/18)	56 kcal (0–210 kcal)

### Interventions

Participants were randomly allocated to one of the following groups:

1. Control: No swaps offered.
2. Swaps: Swaps offered were accompanied by the message: “How about a swap?”
3. Swaps+PACE: Swaps offered were accompanied by the message: “*How about a swap? Save [x] calories* = *[y] min walk”*.

**Fig. 2:**
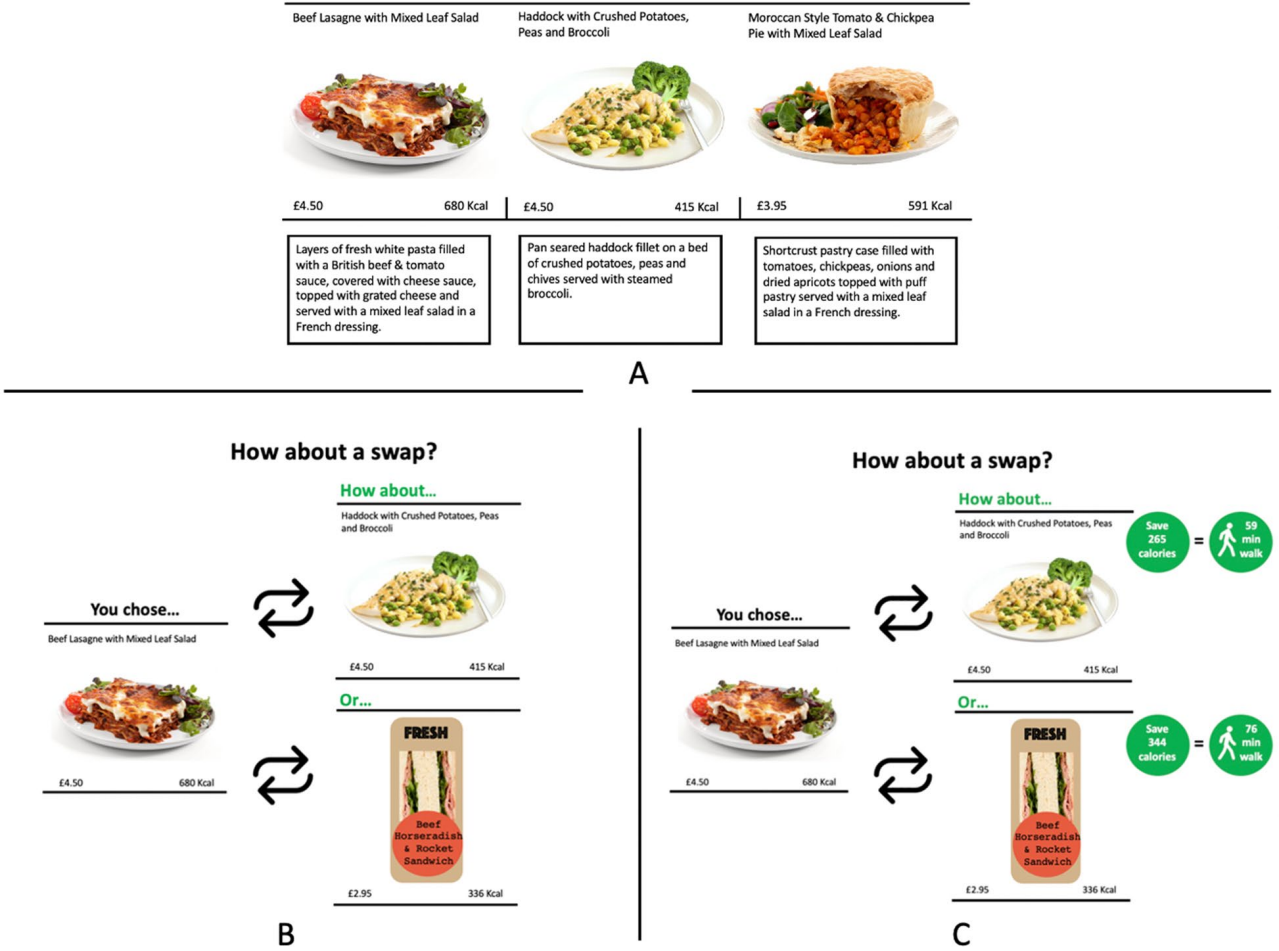
Intervention example: (**A**) Main hot meal menu day 1; (**B**) swaps condition; (**C**) swaps + PACE condition

The energy content in kcal was published beside each option on all menus, meaning that all participants, including the control group, could be aware of the initial calories of all items and had the option to calculate the energy difference between menu items. The price was also presented beside each option on all menus, in all conditions. When swaps were offered, the energy content and the price of the swap item was presented. Figure 2 shows how energy content and price information for menu items and swaps offered was displayed in each condition. Prices were based on the 2020 price list provided to us by a real canteen based in the UK.

### Post-trial survey

After placing their lunch order, participants completed a brief exit survey to explore the acceptability of the intervention and record participant information (sex, age, ethnicity, and education along with height and weight for the calculation of body mass index (BMI). The Scottish Physical Activity Screening Questionnaire (Scot-PASQ) is a validated scale and was used to assess whether participants were meeting physical activity guidelines [12]. A shortened 3-item version (Cronbach’s alpha = 0.81) of the Dietary Intent Scale [13, 14], was used to measure dietary restraint. Acceptability was assessed by asking participants how acceptable they felt it would be for their employer to (a) implement a pre-ordering system for their workplace canteen and (b) offer them swaps for their food choices (only those in the intervention groups). Response options were on a scale from 1 (completely unacceptable) to 5 (completely acceptable). Upon completion, participants were debriefed and reimbursed with £0.50. The protocol (Additional file 1) was implemented with minor changes outlined in Sect. 2.12 Statistical Analysis below.

### The primary outcome

The primary outcome was the total energy (kcal) of items ordered by each of the three groups, controlling for the energy content of the first item ordered.

### Secondary outcomes

- Secondary outcome (a) was the number of swaps accepted (restricted to the groups being offered swaps), controlling for the number of swaps offered.
- Secondary outcome (b) was the proportion of participants ordering a lunch that meets the Public Health England (PHE, 2018) guideline of containing 600 kcal or less in each of the three groups.
- Secondary outcome (c) was Likert scale ratings of intervention acceptability by intervention groups

### Exploratory outcomes

- Exploratory outcome (a) was an interaction analysis examining differences in intervention effects (for the primary outcome) by each of the following variables: sex, age, ethnicity, education, BMI, physical activity level, dietary restraint, and hunger.
- Exploratory outcome (b) was the effect of price difference between the initially selected item and the swap offered by intervention (restricted only to groups being offered swaps) on swap acceptance.
- Exploratory outcome (c) was the acceptance of swaps offered (restricted to the groups being offered swaps), separately for each of the 6 categories: (i) main meals; (ii) jacket potatoes; (iii) soup & sandwiches; (iv) sweet snacks; (v) savoury snacks and; (vi) drinks.
- Exploratory outcome (d) energy ordered controlling for the energy content of the initial item ordered from each menu, separately for each of the 6 categories: (i) main meals; (ii) jacket potatoes; (iii) soup & sandwiches; (iv) sweet snacks; (v) savoury snacks and; (vi) drinks.

### Sample size

We aimed to recruit 2,214 participants. With 80% power, this would allow us to detect a 35 kcal difference at an alpha level of 0.05 (an uncorrected analysis) or a 40 kcal difference at an alpha level of 0.016 (Bonferroni). We applied the Benjamini-Hochberg (BH) correction [15] where the alpha level required was between these two bounds. While a 50 kcal reduction would be a clinically relevant energy reduction for adults [16], we powered the minimum detectable effect size to 35-40 kcal, because we expected the relative effect between the experimental groups to be smaller than the effect between the experimental groups and control. Baseline energy estimates (mean = 423 kcal, *SD* = 236) were taken from a pilot randomised controlled trial conducted in 6 workplace canteens across the UK [17].

### Statistical analysis

A pre-specified statistical plan was published (AsPredicted: 56,358) in advance of the analysis and was followed with minor changes outlined below. Participants had to order at least one food item, not order from all menus (because this was deemed as an implausible lunch order), and checkout to be included in the analysis. The 19 participants (1%) in the intervention groups who were not offered any swaps, because they selected the lowest-energy menu items in all the categories they ordered from were included in the analyses.

- The primary outcome (energy ordered) was analysed using analysis of covariance (ANCOVA), this analysis was pre-registered as ANOVA but ANCOVA was used to control for the energy content of the first item ordered because that was the baseline value of our dependent variable. We controlled for the energy of the first item a participant ordered because we wanted to control for the initial choices participants made but after the first choice their subsequent choices may have been influenced by previous swaps offered.
- The secondary outcome (a) (swap acceptance) was analysed using ordinal logistic regression controlling for the number of swaps offered. This analysis was pre-registered as swap acceptance on a scale from 0–6, however, in the analysis those who ordered from all 6 menus were excluded as improbable values meaning that the scale ranged from 0–5 swaps accepted.
- Secondary outcome (b) (lunch ≤ 600 kcal) was analysed using logistic regression.
- Secondary outcome (c) (intervention acceptability) was analysed using ordinal logistic regression.
- Exploratory outcome (a) (interaction) was analysed using two-way ANCOVA with post-hoc tests controlling for the energy content of the initial item ordered to examine interaction terms between interventions (swaps and swaps + PACE) and the following variables: sex (male vs female), age (equal to or above vs below the median), ethnicity (white vs non-white), education (none/secondary vs higher), BMI (≥ 30 kg/m^2^ vs < 30), physical activity level (meeting guidelines vs not meeting guidelines), dietary restraint (at least the median score vs below the median), and hunger (at least the median score vs below the median). A separate model was conducted for each interaction term.
- Exploratory outcome (b) (overall price difference) was analysed using multilevel logistic regression with an interaction term for intervention and price difference. Analysis was conducted at the ‘swap-level’ and clustering was used to indicate that observations may be correlated within each participant but would be independent between participants. Robust standard errors were used to account for potential heterogeneous effects. For main hot meals where two swaps were offered, price difference was calculated using the price of the swap that was accepted. Where no swap was accepted, the average price difference for the two different swaps offered was used.
- Exploratory outcome (c) (swap acceptance by menu) was analysed using logistic regression. Separate models were run for each of the six menus. The control group was the reference category in all models.
- Exploratory outcome (d) energy ordered by menu. This post-hoc analysis was analysed using ANCOVA, with post-hoc tests controlling for the energy content of the initial item ordered from each menu.

Statistical significance was set at P < 0.05, adjusted with Benjamini-Hochberg (BH) correction [15] for the ANCOVA and regression models (Additional file 1). Mean differences or odds ratios (OR) with 95% confidence intervals (CI) were used to report estimates of comparative effectiveness. Statistical analyses were conducted in Stata (version 16) or SPSS (version 25).

### Results

**Fig. 3:**
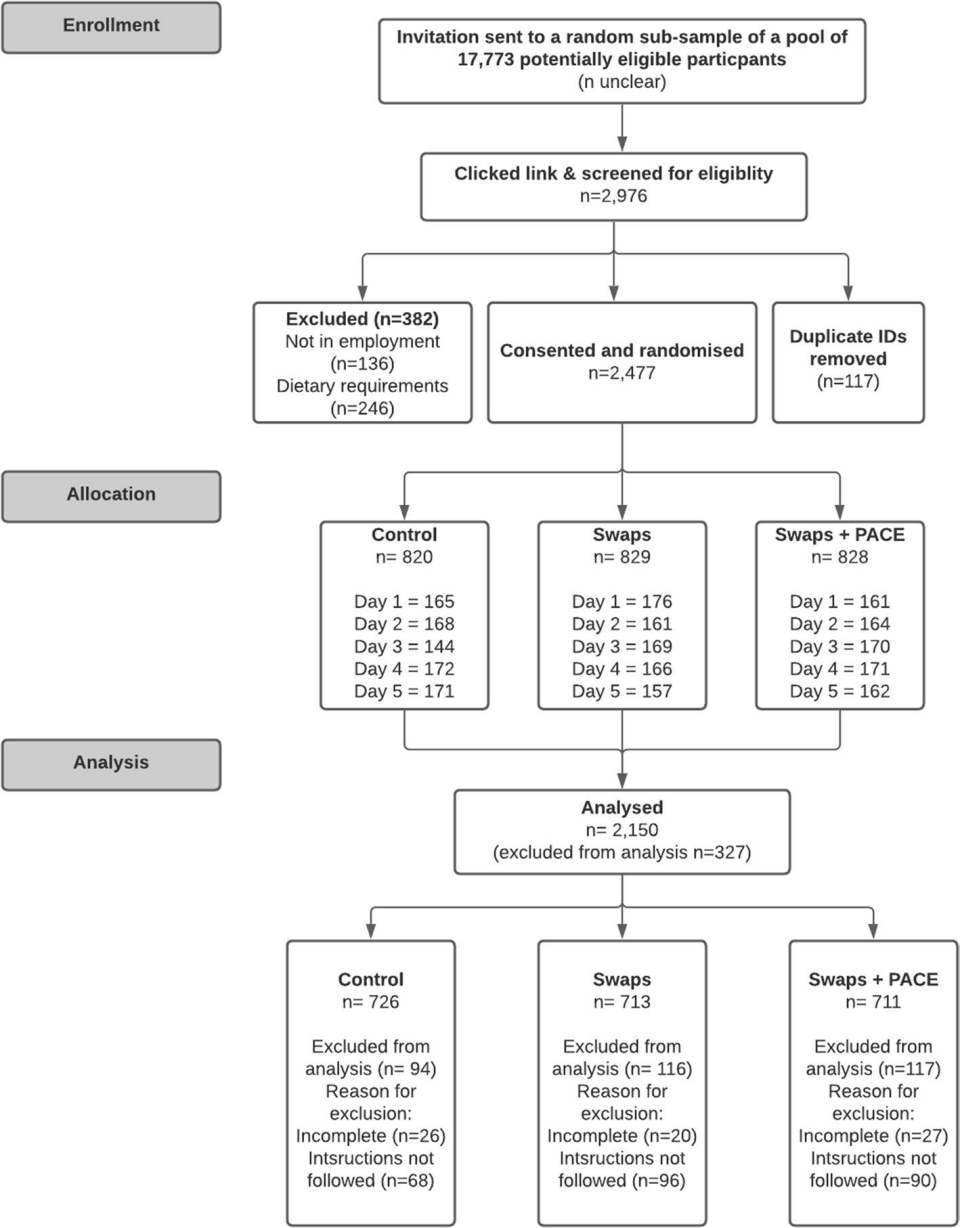
CONSORT flow diagram. Note: Participants who did not follow the instructions had implausible menu orders

Invitations were sent to a random subsample of a pool of 17,773 eligible panel members. Of those invited, 2,477 participants consented and were equally randomised to 1 of the 3 groups. Of those, 2,150 (86.8%) participants followed the instructions, completed the study, and, thus, were included in the analysis (Fig. 3).

Participants were on average 36.8 (*SD* = 11.6) years old. Just over half (54.3%) were female, 80% identified as white, and 51% had completed tertiary-level education (Table 2). Average completion time for the study was 8.2 min (*SD* = 3.18 and attention checks showed high levels of engagement with the task (Additional file 3, Table 1).

**Table 2:** Baseline characteristics of participants. Data are presented as mean ± standard deviation or n (%)

	**Control (*n* = 726)**	**Swaps (*n* = 713)**	**Swaps + PACE (*n* = 711)**	**Total (*n* = 2,150)**
**Age, *years*,**	36.5 ± 11.5	36.8 ± 11.9	37.2 ± 11.5	36.8 ± 11.6
**Sex, *female***	380 (52.3)	387 (54.3)	397 (55.8)	1,164 (54.2)
**Ethnic group**				
White	576 (79.3)	587 (82.3)	560 (78.8)	1,723 (80.1)
Mixed/Other	57 (7.9)	43 (6.0)	47 (6.6)	147 (6.8)
Asian/Black	88 (12.1)	79 (11.1)	102 (14.3)	269 (12.5)
Prefer not to say	5 (0.7)	4 (0.6)	2 (0.3)	11 (0.5)
**Education**				
None to Secondary	352 (48.5)	357 (50.1)	337 (47.4)	1046 (48.7)
Undergraduate degree	238 (32.8)	237 (33.2)	235 (33.1)	710 (33.0)
Graduate & higher	136 (18.7)	115 (16.1)	135 (18.9)	386 (17.9)
Prefer not to say	0	4 (0.6)	4 (0.6)	8 (0.4)
**Anthropometry**				
Weight, kg	77.4 ± 19.0	77.1 ± 18.0	76.9 ± 18.5	77.1 ± 18.5
BMI, kg/m2	26.2 ± 5.6	26.3 ± 5.5	26.3 ± 5.6	26.3 ± 5.6
BMI < 30	577 (79.5)	558 (78.3)	553 (77.8)	1,688 (78.6)
BMI ≥ 30	148 (20.4)	147 (20.6)	153 (21.5)	447 (20.8)
Prefer not to say	1 (0.1)	8 (1.1)	5 (0.7)	14 (0.7)
**Physical activity**				
Meeting guidelines	454 (62.5)	433 (60.7)	443 (62.3)	1,330 (61.9)
Not meeting guidelines	272 (37.5)	279 (39.3)	268 (37.7)	819 (38.1)
Prefer not to say	0	1 (0.1)	0	1 (0.1)
**Hunger score (range: 1–100)**	53.8 ± 24.7	52.0 ± 24.3	53.1 ± 24.0	52.9 ± 24.3
**Dietary restraint (range: 0–15)**	7.5 ± 2.8	7.9 ± 2.8	7.6 ± 2.7	7.7 ± 2.8
**Average energy (kcal) content of first item ordered (i.e. before swaps were offered)**	474 ± 184	459 ± 175	458 ± 172	464 ± 177

*Note: PACE *= Physical Activity Calorie Equivalent; BMI = Body Mass Index. Physical activity: meeting guidelines = 150 min per week of exercise based on the SCOTPAQ screening questionnaire. Higher scores in hunger and dietary restraint indicate higher hunger and restraint, respectively. The average energy (kcal) content of the first item ordered reported is the covariate that is used in the ANCOVA for the primary analysis

### Primary Outcome

**Fig. 4:**
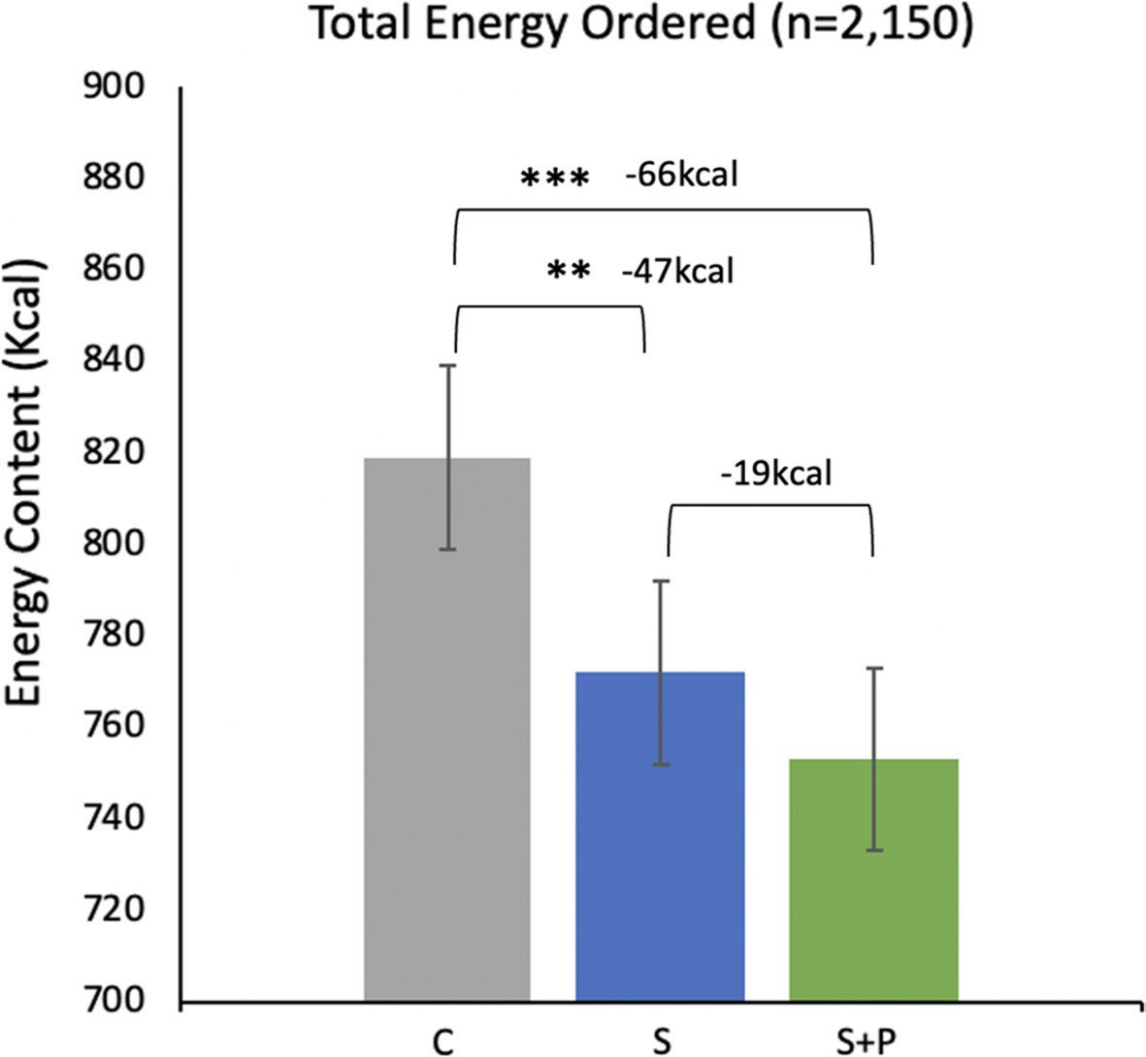
Means and 95% confidence intervals for energy ordered with BH-adjusted p-values: Meets BH threshold^*^, *p* < 0.01^**^, *p* < 0.001^***^. C = control; S = swaps; S + P = swaps + PACE

Participants ordered on average from 3 (*SD* = 0.91) menus. The average energy content of lunches ordered was 781 kcal (SD: 315 kcal, range: 226 to 2,226 kcal). The average energy content of final lunch orders was significantly lower in both intervention groups when compared with control [control mean = 819 kcal]: swaps -47 kcal [95%CI: -82 to -13, *p* = 0.003]; swaps + PACE -66 kcal [95%CI: -100 to -31, *p* < 0.001] (Fig. 4) The difference in the average energy content of final lunches ordered between intervention groups was not statistically significant [-19 kcal, 95%CI: -53 to 16, *p* = 0.591].

### Secondary Outcomes

- *Swap acceptance*
  Almost everyone in the intervention groups (99%) was offered at least one lower-energy swap while placing their lunch order. Table 3 shows the percentage acceptance rate of swaps across the intervention groups. Of the 2,936 swaps offered, 413 (14%) were accepted overall. Table 4 shows the percentage acceptance rate of swaps within each menu across the intervention groups. Compared to the swaps only intervention, the swaps + PACE intervention significantly increased the odds of a swap being accepted, when controlling for the total number of swaps offered [OR: 1.63, 95%CI: 1.27 to 2.09, *p* < 0.001].

**Table 3:** Number (%) of swaps offered and accepted by intervention group (*n* = 1,424)

	**0 Swaps**	**1 Swap**	**2 Swaps**	**3 Swaps**	**4 Swaps**	**5 Swaps**
**Swaps (*n* = 713)**						
*Swaps offered n (%)*	8 (1%)	196 (27%)	291 (41%)	170 (24%)	46 (6%)	2 (< 1%)
*Swaps accepted n (%)*	566 (79%)	128 (18%)	19 (3%)	0	0	0
**Swaps + PACE (*n* = 711)**						
*Swaps offered* *n (%)*	11 (2%)	208 (29%)	284 (40%)	160 (23%)	42 (6%)	6 (< 1%)
*Swaps accepted n (%)*	509 (72%)	165 (23%)	29 (4%)	8 (1%)	0	0

*Note*, Percentages represent the numbers as a proportion of all those in each experimental group

**Table 4:** Number (%) of swaps offered and accepted within each menu by intervention group (*n* = 1,424)

	**Mains**	**Jackets**	**Sandwiches**	**Sweet**	**Savoury**	**Drinks**
**Swaps (*n* = 713)**						
*Swaps offered* *n (%)*	277 (39%)	184 (26%)	290 (41%)	276 (39%)	221 (31%)	234 (33%)
*Swaps accepted n (%)*	52 (23%)	13 (7%)	17 (6%)	42 (15%)	26 (12%)	16 (7%)
**Swaps + PACE (n = 711)**						
*Swaps offered* *n (%)*	295 (41%)	184 (26%)	273 (38%)	261 (37%)	213 (30%)	228 (32%)
*Swaps accepted n (%)*	54 (18%)	32 (17%)	32 (12%)	60 (23%)	26 (12%)	43 (19%)
**Total offered**	572	368	563	537	434	462
**Total accepted**	106 (20%)	45 (12%)	49 (9%)	102(9%)	52 (12%)	59 (13%)

*Note, *Swaps offered is the number (%) of participants in each intervention group that were offered a swap on a given menu. Swaps accepted is the number (%) of participants that accept a swap out of those offered a swap on a given menu
- Meeting public health recommendations
  The proportion of participants who ordered a lunch meeting Public Health England’s energy intake recommendation of ≤ 600 kcal was 26%, 31%, and 36% in the control, swaps, and swaps + PACE groups, respectively. Proportions in the swaps group [OR 1.28, 95%CI 1.02 to 1.61, *p* = 0.0326] and swaps + PACE [OR: 1.57, 95%CI: 1.25 to 1.96, *p* < 0.001] were significantly higher than control. Those in the swaps + PACE pace group were not significantly more likely to meet guidelines than those in the swaps group [OR: 1.22, 95%CI: 0.98 to 1.52, *p* = 0.076].
- Intervention acceptability
  Almost all participants (92%) believed that it would be acceptable for their employer to implement a pre-ordering system for their workplace canteen. Two-thirds (65%) of participants in the intervention groups (i.e., the swaps and swaps + PACE group) believed that being offered swaps for their food choices while pre-ordering would be acceptable (the control group was not asked about the acceptability of swaps). The odds of those in the swaps + PACE group considering the intervention to be acceptable was 1.32 times [95%CI: 1.09 to 1.60, *p* < 0.004] that of those in the swaps only group.

### Exploratory outcomes

**Fig. 5:**
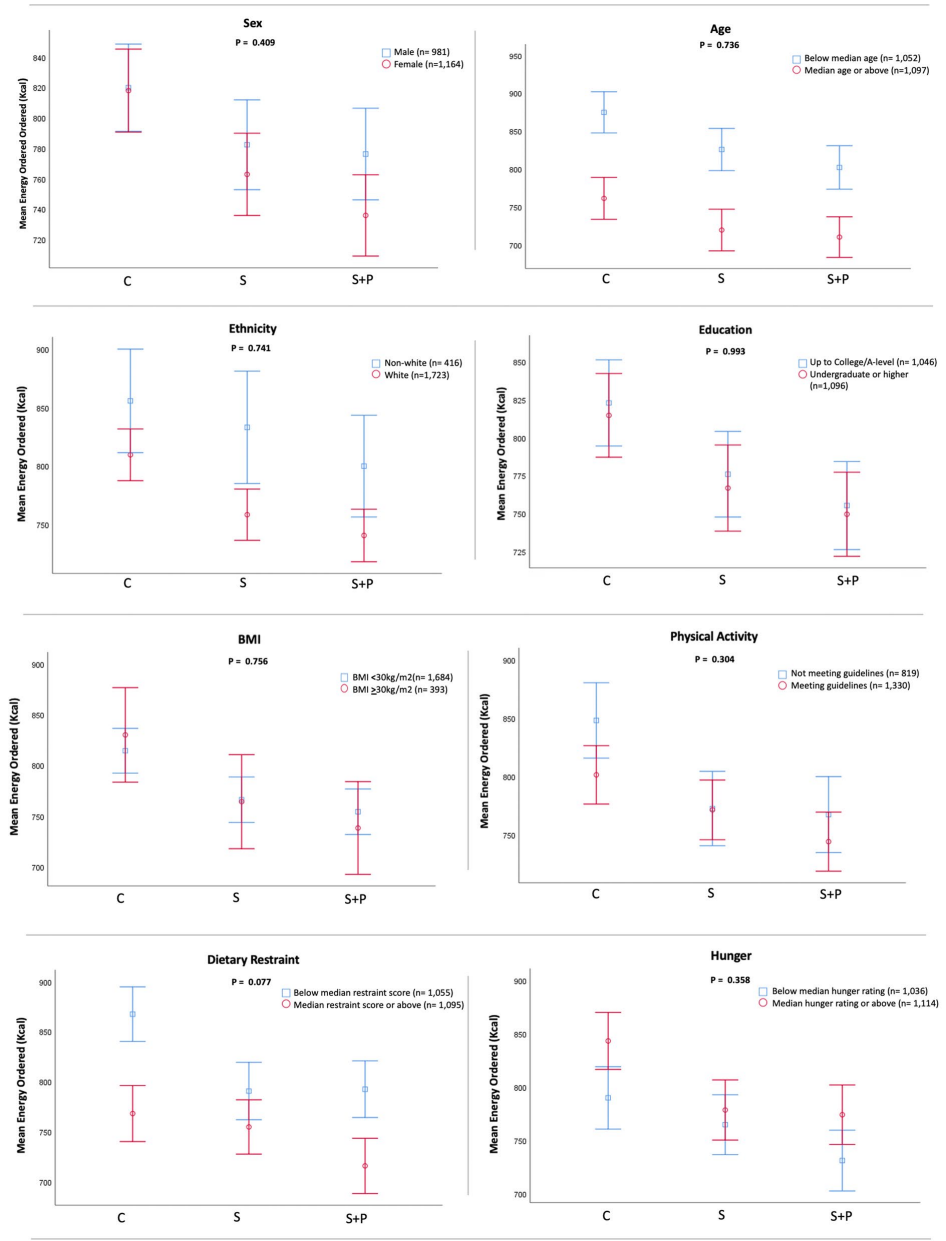
Interaction effect by sex, age, ethnicity, education, BMI, physical activity level, dietary restraint or hunger. Estimated marginal means for final energy ordered controlling for the energy content of the first item ordered with 95% confidence intervals (CIs). C = control; S = swaps; S + P = swaps + PACE. P describes p-values from interaction analysis
- *Swap acceptance and swap price*
  Participants in the control, swaps, and swaps + PACE group spent an average of £5.51 (*SD* = £1.84), £5.29 (*SD* = £1.82) and £5.31 (*S*D = £1.86), respectively on their lunch orders. Table 5 shows the average price of initially selected items and swaps offered for each menu for those in the intervention groups. For 50% of the menus (jacket potatoes, sandwiches and drinks), all swaps offered were either cheaper or the same price as initially selected items. For main hot meals, sweet snacks and savoury snacks, swaps offered were up to £0.55 more expensive than initially selected items.
  For every £1 decrease in swap price, the odds of a swap being accepted increased by 1.85 [95% CI: 1.46 to 2.36, *p* < 0.001]. The interaction analysis demonstrated a greater effect of price difference (between initially selected items and swaps offered) on swap acceptance for those in the swaps group than the swaps + PACE group [p_interaction_ < 0.002] (Fig. 6).

- *Moderation analysis*
  There was no evidence that the intervention effect depended upon sex, age, ethnicity, education, BMI, physical activity level, dietary restraint, or hunger (all p_interaction_ > 0.05, Fig. 5; Additional file 3 Tables 3 – 10).

**Fig. 6:**
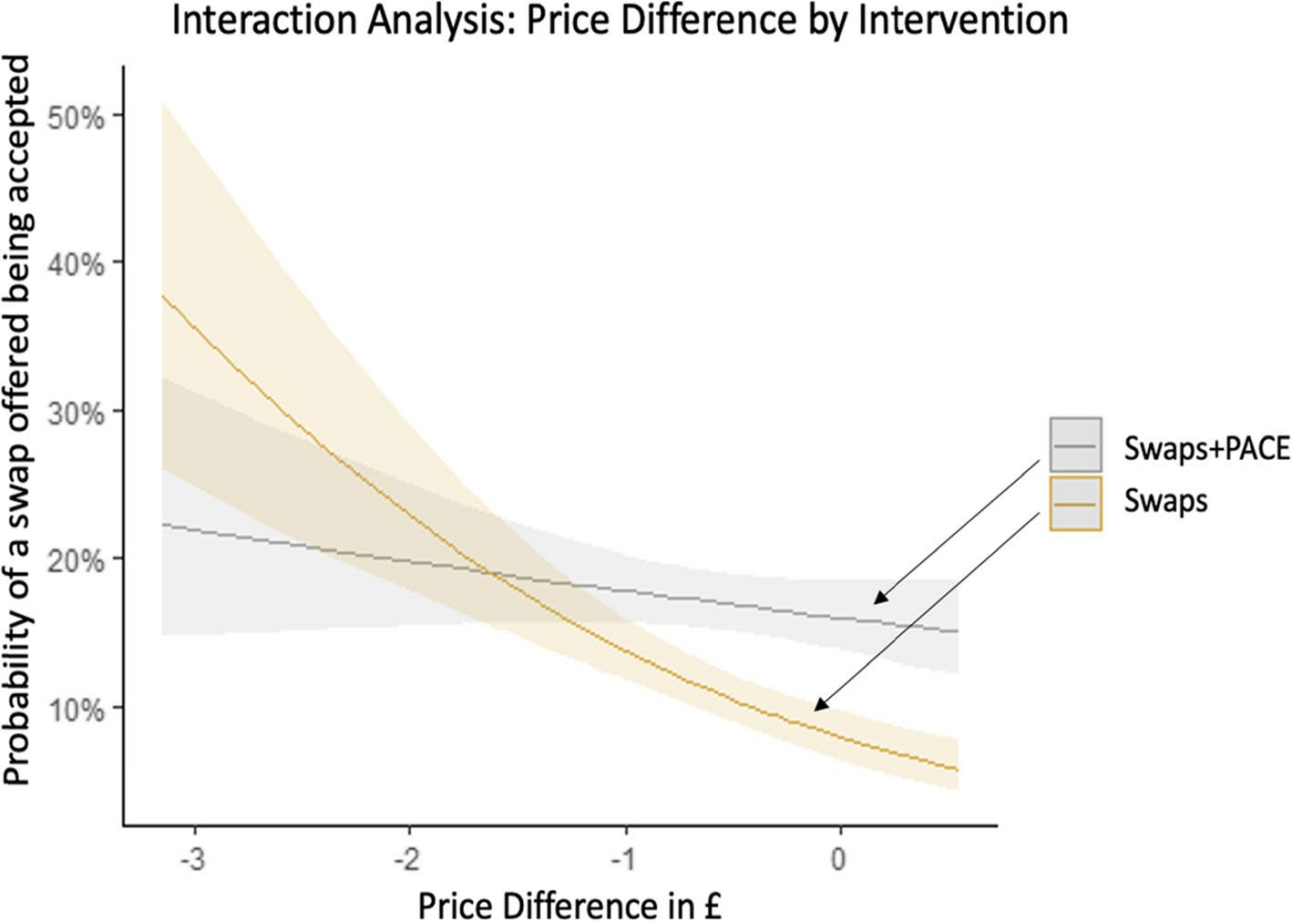
Interaction analysis for the effect of price difference by intervention. Predicted probabilities and 95% CIs

**Table 5:** Prices of initially selected items, swaps offered and price difference for those offered swaps only

	*Initially selected* *£ mean* ± *SD*	*Swaps offered* *£ mean* ± *SD*	*Price difference* *£ mean (95% CIs)*
Mains (*n* = 572)	4.38 ± 0.22	3.03 ± 0.82	-1.36 (-1.43 to -1.29)
Jacket potatoes (*n* = 368)	2.06 ± 0.39	1.45 ± 0.27	-0.61 (-0.65 to -0.58)
Sandwiches (*n* = 563)	2.86 ± 0.26	2.32 ± 0.47	-0.54 (-0.57 to -0.51)
Sweet snacks (*n* = 537)	1.45 ± 0.41	1.23 ± 0.33	-0.22 (-0.25 to -0.19)
Savoury snacks (*n* = 434)	0.74 ± 0.23	1.02 ± 0.19	0.28 (0.25 to 0.31)
Drinks (*n* = 462)	1.35 ± 0.14	0.91 ± 0.21	-0.44 (-0.46 to -0.42)

**Fig. 7:**
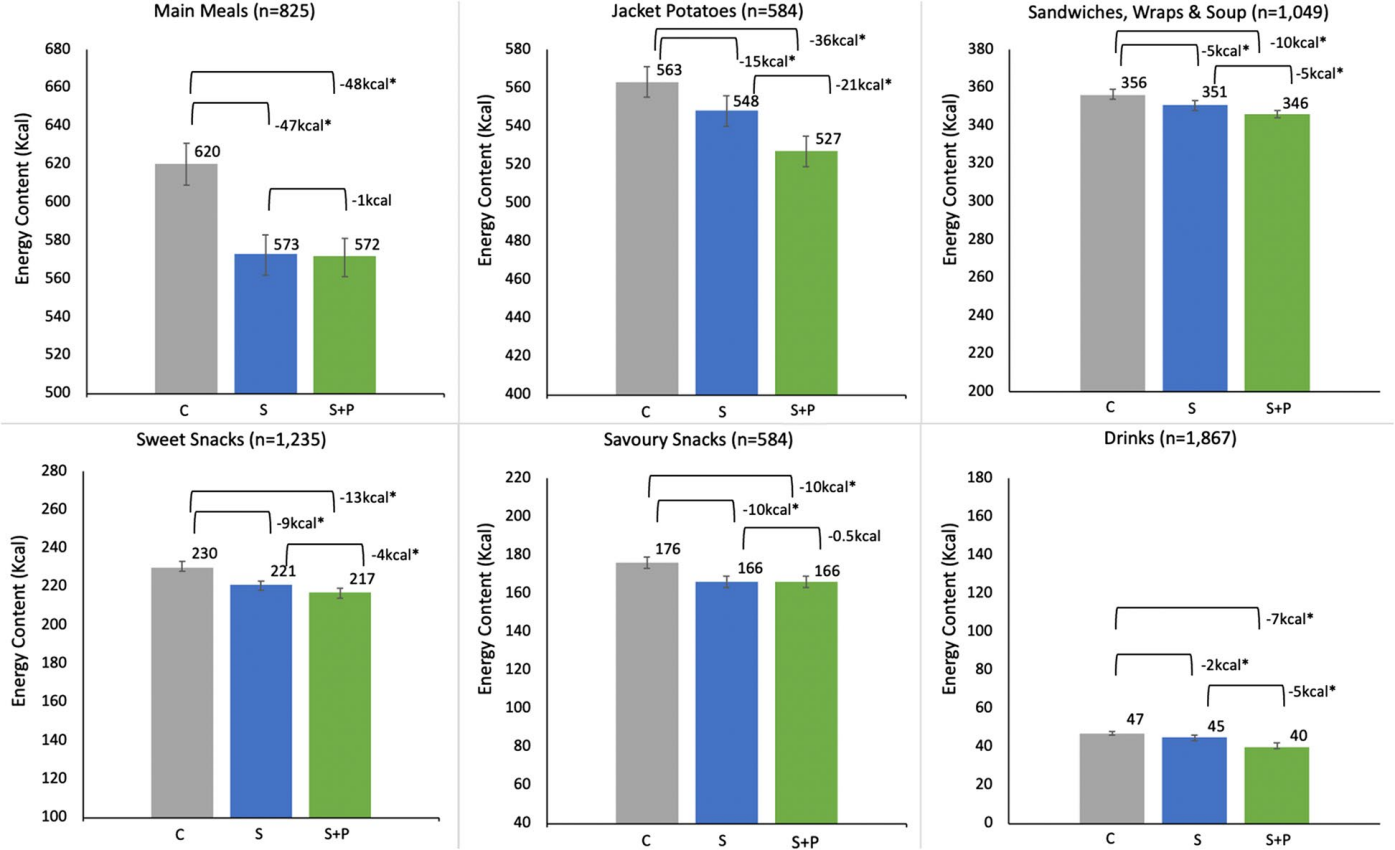
Means and 95% confidence intervals for energy ordered (adjusted for energy of the initially selected item in each category) with BH-adjusted p-values: ^*^Meets BH threshold. C = control; S = swaps; S + P = swaps + PACE. *n* = the total number of items ordered from that category across all 3 arms

*Note*, For price difference, negative values reflect a cheaper swap offered and positive values a more expensive swap offered. *SD* , standard deviation; *95% CIs*, *95% confidence intervals*
- *Swap acceptance and (d) energy ordered by menu*
  Analysis for swap acceptance by menu showed some evidence of differences (Table 4), with participants in the swaps + PACE group being significantly more likely to accept swaps offered for jacket potatoes, sandwiches, and drinks compared to those in the swaps only group (Additional file 3, Tables S11-16). Fig. 7 shows the pairwise comparisons for mean energy ordered (and 95% confidence intervals) in each menu by condition. Significant energy reductions were observed for the intervention groups compared with control on all menus with the largest savings for main meals and jacket potatoes.

## Discussion

The aim of this study was to test the effect of (i) offering lower-energy swaps, and (ii) offering lower-energy swaps with a PACE message on the total energy of items pre-ordered for lunch within the context of an experimental online workplace canteen. Offering lower-energy swaps significantly reduced the energy content of lunches pre-ordered compared with not offering swaps. Although accompanying swaps offered with PACE information significantly increased swap acceptance relative to when this information was not provided, it did not significantly reduce energy pre-ordered. Both interventions resulted in significantly more participants ordering lunches that met public health recommendations of containing ≤ 600 kcal, compared with the control group. Acceptability was high overall, but significantly higher among those presented with PACE information when swaps were offered.

### Interpretation and implications

To our knowledge, this study is the first to provide experimental evidence that offering lower-energy swaps for lunchtime meals selected through online canteens may effectively reduce energy of foods ordered. Previously, studies have focused on demonstrating the ability of swap-based interventions to improve the healthfulness of items purchased when shopping online for groceries [3, 4, 18]. One reason for this is that similarity between swaps offered and original selections is thought to increase the likelihood of swap acceptance [19]. Grocery shopping usually entails the purchasing of many discrete products for which very similar, more healthful alternatives are readily available. By contrast, choices made in canteen environments are typically between a smaller range of prepared meals for which close alternatives are usually unavailable.

Although providing PACE information when offering swaps is likely to require some additional investment from organisations especially in canteens where menus change daily, our results indicate that adding PACE information significantly increases swap acceptance and intervention acceptability. The higher acceptability ratings among those in the swaps + PACE group may be explained by evidence that intervention acceptability is influenced by the extent to which participants understand the intervention and how it works [20]. In the swaps condition, the reason that swaps are being offered may not be instantly apparent to participants, whereas in the swaps + PACE condition, the message accompanying swaps offered makes salient to participants that swaps are being offered because they are lower in energy. This information may have aided intervention understanding among the swaps + PACE group, thereby increasing intervention acceptability. Although our analysis was not powered to detect differences between individual menus, our results suggest that offering swaps may be more effective for certain menus such as main meals, jacket potatoes, and sweet snacks. Canteens in which menus change regularly and are not planned well in advance could potentially minimise the effort required of offering swaps and PACE information for ever-changing menus by implementing automated versions of these interventions for specific menus only. PACE information could be provided for menu items that change less regularly or for which messaging has the largest effect. For example, there was evidence of larger energy reductions and greater swap acceptance when PACE information was provided for the jacket potatoes menu compared with offering swaps only.

Exploratory analysis did not point to any notable differences in the intervention having differential effects by participant characteristics. This is important given that interventions relying on individual decision making and agency are often criticised for potentially increasing health inequalities [21]. One potential exception to this is the observation that unrestrained eaters seemed to tend towards being more influenced by the intervention than restrained eaters. While this analysis was underpowered and the results were not statistically significant, this trend might suggest that offering swaps would potentially be most effective for those most in need of intervention (i.e. consumers with lower dietary restraint). The high ratings for intervention acceptability overall indicate that interested organisations could trial the intervention without concerns that their employees would be dissatisfied with this change. Employee surveys highlighting the link between the provision of worksite food services and worker wellbeing [22] suggest that, if implemented correctly, canteen pre-ordering services may be both acceptable and welcomed [6]. Two-thirds of those offered swaps felt that this would be an acceptable feature of a workplace pre-ordering website. This is similar to acceptability ratings reported in previous studies [3, 5] and echoes recent research indicating that canteen customers expect a trend towards the increased availability and promotion of healthy dishes [23]. The findings of this study are especially applicable with the return to the workplace after the first waves of COVID-19, where pre-ordering systems are more likely to be implemented.

Most swaps offered in this study were cheaper than their high-energy counterparts, because they were either vegetarian or had fewer extras (e.g., swaps for jacket potatoes with beans and cheese was jacket potatoes with beans only). Our analysis indicates that offering cheaper swaps significantly increases the likelihood that a swap will be accepted. Recent research supports the idea that even small price reductions (£0.20), can influence food purchasing behaviours in the canteen environment [24]. It seems that when the price difference between initially selected items and swaps offered is small or the swap offered is more expensive, PACE messaging increases swap acceptance, but as the swap becomes cheaper the effect of the price difference becomes more impactful for the swaps only group. Although price was not directly manipulated in this study (i.e., participants were not randomised to receive cheaper or more expensive swaps), the interaction analysis indicated that both swap + PACE and swap only accompanied by a price reduction could possibly be equally effective in terms of swap acceptance for a reasonably implemented price reduction of approximately £1–1.5. Swap acceptance is lowest when swaps alone are offered, however, adding either price reduction or PACE increases the probability of swap acceptance. To maximise efficacy, organisations should aim to offer cheaper lower-energy alternatives. Where this is not possible, the addition of PACE information might aid swap acceptance. Further research is required to examine, in detail, the relative effect of price difference on swap acceptance, ideally in a setting where participants are making actual purchases with their own money. It is also important to note that as well as being offered swaps (some of which were cheaper), all participants were provided with energy information. The findings of the current study support the view that multiple strategies (swaps, price incentives, calorie disclosures) are simultaneously needed to change dietary behaviour.

### Effectiveness of offering swaps with and without PACE information compared to other interventions

Only 14% of lower-energy swaps were accepted overall. When compared with experimental online supermarket studies, this mirrors the acceptance rate for swaps lower in saturated fat [3] but is smaller than the 34% acceptance rate for swaps lower in salt [4]. Offering swaps for lunch choices, nevertheless, significantly reduced energy ordered by between 47 and 66 kcal on average. When considering the pre-post figures, effect sizes observed appear to be of a similar magnitude to studies testing PACE labels in canteens (-40 kcal) [25], and slightly smaller than other canteen-based interventions targeting energy reduction by reducing portion sizes (-74 kcal) [26], and multi-component interventions such as increased availability of lower-energy options and price discounts (-70 kcal) [27]. However, in order to isolate the effect of offering swaps and offering swaps with PACE information in the current study, all participants, including the control group, were provided with energy information for menu items. Previous research testing the effect of placing PACE labels on products sold in real-world worksite canteens (where energy information is not widely available) reported an average energy reduction of 40 kcal when these labels were present compared to when they were absent [25], but found no significant difference between providing PACE information and calorie-only information (-38 kcal). In the current study, energy information was displayed for all groups. Compared with the control condition, offering swaps significantly reduced energy ordered by 47 kcal on average. A Cochrane review on energy labelling suggests that calorie-information on menus also reduces energy purchased by 47 kcal on average compared to when no labelling is provided [28]. It is, therefore, possible that offering swaps would yield a larger energy reduction than the 47 kcal reported here, when compared with orders placed directly from a canteen where energy information is unavailable for most food items.

### Strengths and limitations

This study used a randomised design and recruited a large sample of employed adults that broadly matched the distribution of the UK population in terms of sex, ethnicity, and education. Participants were randomised to see 1 of 5 different menus, meaning that swaps were offered for 15 different main hot meals. This menu variety helps to increase the generalisability of our findings. By partnering with a real-world company and simulating a pre-ordering website using their canteen menus, this study was able to test the effect of offering lower-energy swaps for lunch time meals in a similar manner to how choices would be made when using an online canteen in real life. Qualitative research with employees of the partner organisation informed swap choices and intervention delivery [29].

The primary limitation of this study is its hypothetical nature. Participants made imaginary choices and were not required to spend their own money. The experimental nature of this study means that effect sizes observed in real-world settings may be smaller than those reported here [30]. We attempted to minimise this issue by asking participants to make choices that were in keeping with their usual purchasing behaviours, and by using the menus of a real-world canteen. Although setting the minimum energy reduction for swaps offered at 50 kcal was based on evidence which suggests that this would be a clinically relevant reduction [11], it meant that only a limited choice of swaps were available for each item. The appeal of these swap items would affect the real-world effectiveness of the intervention. Intervention acceptability was assessed in principle (i.e., without participants having tasted the swaps they accepted). It is possible that if participants did not enjoy their swaps, acceptability ratings would be lower. Given the nature of the sample (professional survey takers), it is possible that the results may not entirely reflect the behaviours of the general population. While the demographic characteristics of panel members sampled broadly matched those of the general UK population, little is known about the generalisability of the study findings outside of the UK. Self-reported height and weight measures to calculate BMI may also have been influenced by social desirability bias [31]. Although the measure of dietary restraint was based on a validated scale [14], due to time constraints, a shortened unvalidated version was used, which still maintained a high Cronbach's alpha (α = 0.81) (as detailed on page 12 of Additional file 1). Our analysis was in available cases. Although imputing data for non-completers may have slightly attenuated our estimates, the proportion of missing data was relatively small (13%) and therefore any such biases are unlikely to affect the interpretation of the results. Finally, total energy intake at baseline may have been an effect modifier, but we did not measure it due to well-known limitations of existing methods. However, we did not find any evidence that the effect depended upon hunger, dietary restraint, physical activity level, or BMI (a reasonable proxy for energy balance), so such effect modification of total energy intake, if it exists, is not likely to substantially modify intervention effects. As well as targeting energy consumption, the promotion of broader healthy dietary patterns (e.g., reducing the consumption of ultra-processed food) is important and should also be a consideration for future studies in this space.

## Conclusion

Offering lower-energy swaps has the potential to reduce the energy of lunches ordered through online canteens. Accompanying swaps offered with PACE information did not change the energy of lunches ordered compared to offering swaps alone, but it increased the swap acceptance rate and intervention acceptability. Future work should test the effect of a pre-ordering website that prompts users with swaps on energy ordered in real-world canteens over extended periods of time to observe sustainability of any intervention effects.

## Supplementary Information

**Additional file 1.**

**Additional file 2.**

**Additional file 3.**

**Additional file 4.**

## Data Availability

The datasets used and/or analysed during the current study are available from the corresponding author on reasonable request.

## Abbreviations

ANCOVA: Analysis of Covariance
BH: Benjamini-Hochberg
BMI: Body Mass Index
CI: Confidence Interval
Kcal: Kilocalories
OR: Odds Ratio
PACE: Physical Activity Calorie Equivalent
REDCap: Research Electronic Data Capture
Scot-PASQ: Scottish Physical Activity Screening Questionnaire
SD: Standard Deviation
