# Supplementary material for: Strategies to reduce the energy content of foods pre-ordered for lunch in the workplace: a randomised controlled trial in an experimental online canteen
Source: Int J Behav Nutr Phys Act. 2022 May 12;19:54. doi: 10.1186/s12966-022-01257-5 (PMC9096740; doi:10.1186/s12966-022-01257-5)
Supplement: Supplementary file 1 — Additional file 1. [file 12966_2022_1257_MOESM1_ESM.pdf]

Additional File 1 – Study Protocol

Study Title: Strategies to reduce the energy content of foods pre-ordered for lunch in the workplace: a randomised controlled trial in an experimental online canteen

Ethics ID Number: 12861/005

Primary Investigator: Sarah Breathnach
sarah.breathnach.17@ucl.ac.uk , 07873758162
Research Department Behavioural Science and Health
Faculty of Epidemiology & Public Health
University College London

Investigators: Dr Clare Llewellyn, Dr Phillippa Lally
Research Department Behavioural Science and Health
Faculty of Epidemiology & Public Health
University College London

Dr Dimitrios A Koutoukidis
Nuffield Department of Primary Care Health Sciences
University of Oxford
NIHR Oxford Biomedical Research Centre, Oxford University Hospitals NHS Foundation Trust, Oxford, UK

Dr Alex Sutherland
Behavioural Insights Team
4 Matthew Parker Street
SW1H 9NP, UK

Sponsor: University College London

Funder: The Behavioural Insights Team

Conflicts of interest: None declared.

Table of Contents

1. SYNOPSIS 2
2. BACKGROUND & RATIONALE 3
    2.1 Study aims 4
3. STUDY DESIGN & SETTING 4
4. PARTICIPANT IDENTIFICATION AND RECRUITMENT 4
5. RANDOMISATION & BLINDING 5
6. STUDY PROCEDURES 7
    6.1 Online ordering task 7
    6.2 Swaps offered 7
    6.3 Intervention 10
    6.4 Pre-task questions & exit survey 11
7. STATISTICS AND ANALYSIS 13
    7.1 Sample Size 13
    7.2 Outcome Measures 14
    7.3 Statistical Analysis 15
8. DATA MANAGEMENT 17
9. QUALITY CONTROL & QUALITY ASSURANCE PROCEDURES 17
10. ETHICAL CONSIDERATIONS 18
11. FINANCE & INSURANCE 18
12. PUBLICATION POLICY 19
13. REFERENCES 19

Appendix A: Screening survey & pre-task questions 23

Appendix B: Task instructions 23

Appendix C: Exit survey & debriefing text 24

Appendix D: Full menus 24

1. SYNOPSIS

**Study Title**	Strategies to reduce the energy content of foods pre-ordered for lunch in the workplace: a randomised controlled trial in an experimental online canteen	
**Ethics ID no.**	12861/005	
**Study Design**	3-Arm Randomised Controlled Trial	
**Study Participants**	Prolific Academic panel members	
**Planned Sample Size**	2,214 (738 per arm)	
**Planned Study Period**	December 2020 - March 2021	
	**Objectives**	**Outcome Measures**
**Primary**	To test;<br><br>(a) the effect of the interventions on total energy of ordered items.	(a) the difference in the total energy (kilocalories/kcal) of items ordered between groups (control vs swaps; control vs swaps+PACE; swaps vs swaps+PACE).
**Secondary**	To investigate;<br><br>(a) the effect of the different interventions (Swaps and Swaps + PACE groups only) on the number of swaps accepted.<br><br>(b) the effect of the interventions on the proportion of participants ordering a lunch that meets the Public Health England's (PHE) guideline of containing 600 kcal or less.<br><br>(c) the difference in the acceptability of (i) the pre-ordering system and (ii) offering swaps between groups.	(a) the difference in the number of swaps accepted between intervention groups; swaps and swaps+PACE, controlling for the number of swaps offered.<br><br>(b) the difference in proportions of participants ordering a lunch that meets the PHE's guideline of containing 600 kcal or less (control vs. swaps; control vs. swaps+PACE; swaps vs. swaps+PACE).<br><br>(c) the difference in Likert scale ratings of acceptability between interventions (i) control vs. swaps, control vs. swaps+PACE; (ii) swaps vs. swaps+PACE.
**Exploratory**	To investigate;<br><br>(a) whether the effect of the interventions differ by:ex, age, ethnicity, education, BMI, physical activity level, dietary restraint and hunger.	(a) interaction analysis of the primary outcome by each of the following separately: sex, age, ethnicity, education, BMI, physical activity level, dietary restraint and hunger.

	(b) the effect of price difference on swap acceptance (swaps and swaps+PACE groups only).<br><br>(c) acceptance of swaps offered between Swap and Swaps + PACE groups for each of the 6 menu categories separately (i) main meals, (ii) jacket potatoes, (iii) soup & sandwiches (iv) sweet snacks (v) savory snacks and; (vi) drinks.	(b) among those offered a swap, the effect of price difference between the initially selected item and the swap offered on swap acceptance (analysis at the swap-level).<br><br>(c) Accceptance of swaps offered between swap and swaps+PACE groups separately for each of the 6 menus.

2. BACKGROUND AND RATIONALE

A positive energy balance leads to weight gain. At population level, this primarily occurs through excess energy intake (Church & Martin, 2018). The development of strategies to reduce population energy intake is therefore essential to halting the rise in obesity (PHE, 2018).

Prompting consumers to swap their initial selections for healthier alternatives while shopping may help bring energy intake into line with public health recommendations. Swap-based interventions have been tested in experimental settings and the results show reductions in both the saturated fat (Koutoukidis et al., 2019) and salt (Payne Riches et al., 2019) content of grocery baskets. More recently, offering swaps was shown to reduce the energy content of snacks and drinks ordered in an experimental online canteen (Breathnach, et al. 2021). This study also found that accompanying swaps offered with physical activity calorie equivalent (PACE) information, indicating the amount of energy contained in a food or drink and the amount of physical activity that would be required for it to be expended, significantly increased the likelihood that a swap offered would be accepted when compared to offering swaps with no specific information. The provision of PACE information also increased intervention acceptability ratings. These findings indicate that providing easily interpretable or tangible information when offering swaps for snacks or drinks increases their acceptance.

Similar to online supermarkets, pre-ordering websites for canteens provide a platform for intervention delivery and are likely to become increasingly popular (Migliavada et al., 2021). However, little is known about whether lower-energy swaps offered across a full canteen menu including items such as hot meals or sandwiches would be accepted. And if swaps are

accepted, whether consumers compensate for reductions (e.g. by ordering more items and thus more energy) across their whole meal.

2.1 Study Aims

The aim of this study is to test the effect of (i) offering lower-energy swaps, and (ii) offering lower-energy swaps with a PACE message on the total energy of items ordered for lunch within the context of an experimental online workplace canteen. We will examine whether there is an added benefit of providing a tangible message with the prompt to swap by comparing the energy ordered by those offered swaps with no information and those offered swaps with PACE information.

3. STUDY DESIGN & SETTING

This is a 3-armed randomised controlled trial of an intervention to reduce energy of items ordered in a simulated online canteen developed using REDCap (Research Electronic Data Capture). REDCap is a secure, web-based application designed to support data capture for research studies, providing: 1) an intuitive interface for validated data entry; 2) audit trails for tracking data manipulation and export procedures; 3) automated export procedures for seamless data downloads to common statistical packages, and 4) procedures for importing data from external sources (Harris et al., 2009). The design of the platform is based on a custom-made simulated online supermarket platform, used in previous studies (Koutoukidis et al., 2019; Forwood et al., 2016) adapted to emulate an online canteen for pre-ordering.

An online canteen pre-ordering system is a website which displays the canteen's menu and allows employees to place their lunch order in the morning for collection later that day. For this study, participants will be able to hypothetically order their lunch from 6 menus containing a selection of main hot meals (n=3), jacket potatoes (n=10), soup & sandwiches (n=15), sweet snacks (n=18), savoury snacks (n=20), and non-alcoholic drinks (n=18) based on the menus of a real-world workplace canteen. Participants will be able to order what they like from the menus. In the real-world canteen, main hot meal options change on a daily basis. To reflect this, participants will be randomly assigned to view the main hot meals (n=3) for one of five different menus. This means there will be a total of 15 experimental groups (5 menus x 3 arms). See Appendix D for full menus.

4. PARTICIPANT IDENTIFICATION AND RECRUITMENT

4.1. Participants, Screening and Eligibility Assessment

Participants will be recruited through Prolific Academic, an online participant sourcing platform in January/February 2021. Prolific Academic has a large panel of members that is

broadly representative of the general UK population in terms of gender, age, location, and income.

Eligible participants can access the study through their Prolific Academic dashboard or are invited directly through an email notification from Prolific Academic. Participants are given some high-level information about the study to help them decide if they want to proceed. E.g. Title: “5-minute lunch ordering”. Description: “The aim of this study is to test a new online ordering system for a workplace canteen. We ask you to imagine that you use this system to place a lunch order from our menus.” To be eligible for the study, participants need to be ≥18 years, a UK resident, speak English fluently and be in full or part-time employment.

The above criteria will ensure that the sample matches the target population of the intervention. People following a medically prescribed diet or restricted diet such as a vegetarian, vegan, sugar-free or gluten-free diets are not eligible as this would influence the appropriateness of swaps offered.

4.2. Informed Consent

A link to the study will appear on the Prolific Academic dashboard of potentially eligible participants. Eligible participants will be able to read the information sheet which will provide a broad description of the study, which will avoid stating the explicit aims to ensure participants are blinded. Participants will provide consent electronically and complete the study via the link published on Prolific Academic.

Participants will be given as much time as they need to consider the information, and the opportunity to question the Investigator (via email). Electronic informed consent will then be obtained by means of clicking on a tick box. Once consent is obtained participants will be automatically randomised (see section 5 below).

4.3. Discontinuation/Withdrawal of Participants from Study

Each participant has the right to withdraw from the study at any time. We will record withdrawal but the reason for withdrawal will not be recorded. We will check for differential attrition to ensure that the likelihood of drop-out is not predicted by treatment allocation. Participants will only be paid if they complete the study. This is outlined in the consent form.

5. RANDOMISATION & BLINDING

Simple randomisation will be performed using Predictiv (Behavioural Insights Team LTD, 2021). Participants will be randomised to both a trial arm (1 of 3) and a menu (1 of 5), meaning that participants will be evenly allocated to 1 of 15 groups (Table 1). To do this, the platform will allocate eligible participants a random integer between 1 and 15 representing

the 15 conditions. To ensure balance, the algorithm ranks the conditions (1-15) based on the number of participants previously allocated to each and allocates the next participant to one of the 7 least used conditions (Table 1a).

Table 1: Randomisation allocation schedule.

**Menu 1**	**Menu 2**	**Menu 3**	**Menu 4**	**Menu 5**
1. Control	4. Control	7. Control	10. Control	13. Control
2. Swaps	5. Swaps	8. Swaps	11. Swaps	14. Swaps
3. Swaps + PACE	6. Swaps + PACE	9. Swaps + PACE	12. Swaps + PACE	15. Swaps + PACE

Table 1a: Example of self-balancing randomisation procedure. n indicates the number of participants already allocated to each condition. The red-shading indicates the "least used conditions" at this point in the randomisation.

**Condition**	**n**	**Condition**	**n**	**Condition**	**n**
Condition 1	200	Condition 6	209	Condition 11	214
Condition 2	201	Condition 7	204	Condition 12	207
Condition 3	207	Condition 8	203	Condition 13	210
Condition 4	202	Condition 9	206	Condition 14	205
Condition 5	211	Condition 10	202	Condition 15	199

The randomiser will split the list into most used conditions (i.e. conditions: 3, 5 ,6, 9, 11, 12, 13, 14 in the example table) and least used conditions (i.e. conditions: 1, 2, 4, 7, 8, 10, 15). In our case, where there is an uneven number of conditions the list will be split by the bottom half rounded down, meaning that the next participant will be allocated to one of the 7 least used conditions.

Depending on the number assigned, participants will be sent to a separate web page on Predictiv with a link that corresponds to one of the 15 'surveys' (i.e. conditions) on REDcap. Allocation concealment will be achieved as participants will be recruited by Predictiv and will be directed to Predictiv for automatic randomisation. Participants will not be aware that there are other groups who will see different information.

Investigators will not be blinded to intervention allocation, but they will not be able to manipulate any study parameter following the initial study set up, as all study procedures are taking part in the online platform. The outcome assessment is blinded, as it happens automatically in the online platform. The chief Investigator will analyse the data and will not be blinded to intervention allocation. Due to the nature of the intervention, it will not be

possible to blind participants to the intervention. However, participants will only be aware of the trial arm that they are exposed to and will be unaware of the other trial arms and the primary aim of the trial.

6. STUDY PROCEDURES

6.1 Online ordering task & exit survey

Following randomisation, participants will be redirected to REDCap where they will be asked to indicate their current subjective feeling of hunger using the visual analog scale (VAS) (see Section 6.4 for details). After this, participants will be asked to imagine they work for a company that has a pre-ordering website for their canteen. Participants will be asked to order their lunch for the day using the website. They will be able to order from as many menus as they wish but will be asked to make choices that are in keeping with what they would typically have for lunch during their working day (see Appendix B). Participants will be offered swaps to alternative products if a suitable swap is available (see section 6.2 for swap criteria). Participants can decline or accept the swap for all menus except the main hot menu by clicking either "*No, I will stick with my choice*" or "*Yes, I would like to swap*". For the main hot menu, where two swaps are offered, participants can decline or accept the swaps offered by clicking one of the following 3 options: "*No, I will stick with my choice*", "*Yes, I would like to swap to [name of swap 1]" or "Yes, I would like to swap to [name of swap 2]."* Once participants have made their selections, they will be asked to complete a short exit survey (Appendix C). This will include (i) an attention check (ii) questions on the acceptability of the intervention and (iii) a demographic characteristics questionnaire. Upon completion, participants will be debriefed and reimbursed with £0.50 for their participation in line with Prolific Academic guidelines.

6.2 Swaps offered

For all items, except the main dishes, participants will be offered 1 suggested swap immediately after making each selection. Swaps offered will be from the same menu as the original selection to ensure highest possible similarity.

The main hot dishes are dissimilar to provide variety, therefore offering a swap from within the same menu results in offering a dissimilar product. To maximise the potential acceptance of swaps offered, 2 swaps will be offered for each main dish. In most cases, the first swap offered will be the lowest energy main and the second swap offered will be either a jacket potato or an item from the sandwich and panini menu, which is judged (by the research team) to be similar to the originally selected item. Similarity will be based on the main protein source in the dish. Where possible, the main protein source will be matched. For example, a chicken sandwich would be offered as a swap for a chicken curry. Figure 1

outlines the decision process for offering swaps for main dishes and Table 2 outlines the criteria for swaps offered according to each menu. To qualify as a swap in a menu, the alternative has to contain at least 50 kcal less than the originally selected item, because a minimum of 50kcal reduction per-person per-day has been identified as being clinically relevant (Hill, Wyatt, Reed, & Peters, 2003).

A registered dietitian analysed the energy content of menu items using recipes provided to us by our partner canteen's catering company and the nutritional information published on the supplier's website. Recipes contained the ingredient name, quantity in grams (or millilitres), and the number of portions the recipe provides. The catering company also provided us with energy information for all of the pre-packaged food and drinks. Energy information will be presented for all items, to all groups.

Figure 1: Flow diagram outlining the criteria for swaps offered for main dishes.

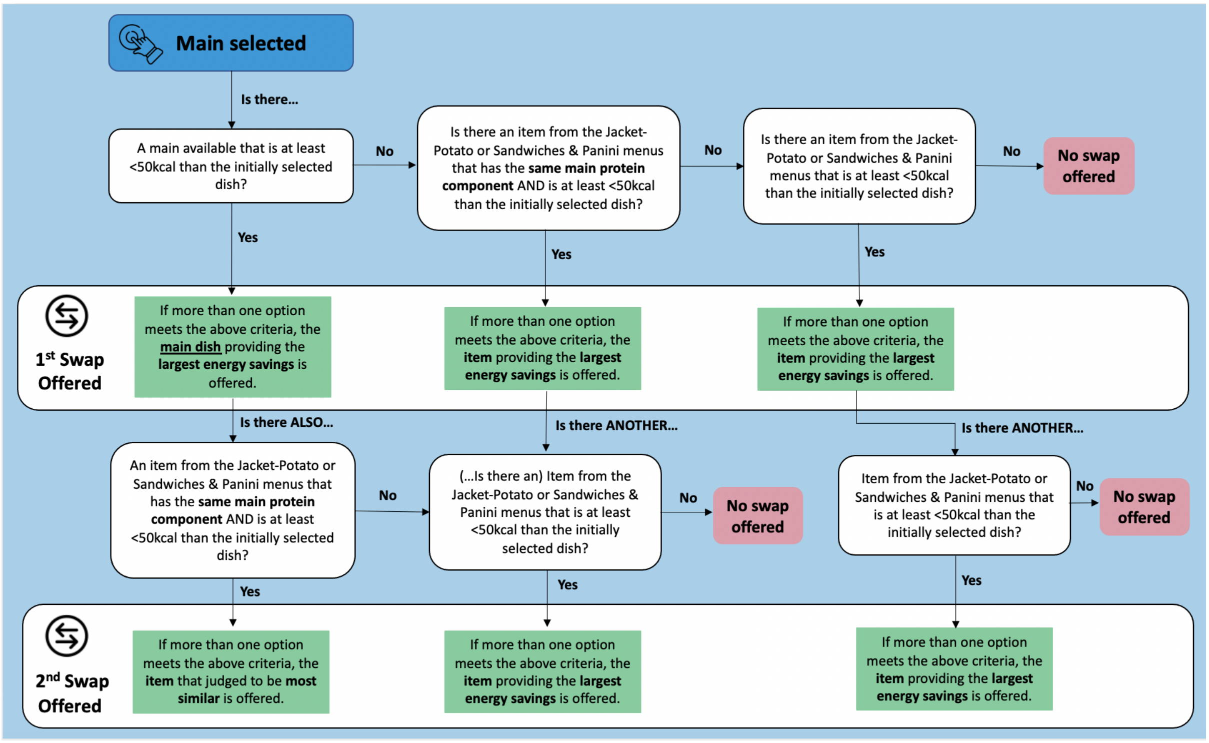

Note: If the main protein was vegetarian (e.g. a vegetable, bean, pea or lentil) the protein was not always the same. For instance, in some cases where the main component was chickpea a bean-based meal was offered as a 'similar' swap.

Table 2: Criteria for swaps offered by menu

**Menu**	**Criteria**	**Example of swap offered**	**% Swaps available**
Main meals (n=3)	See Figure 1		100%

Jacket Potatoes (n=10)	- Swaps offered are as close in nature to the originally selected item as possible.<br>- Swaps offered are at least 50kcal less than the originally selected item.<br>- For jacket potatoes, swaps offered usually involve the removal of one of the high energy toppings.<br>- For sandwiches, the swap offered is the sandwich that has the same protein and is at least 50kcal lower. If not possible, the swap offered is the sandwich lowest in terms of calories. If not possible, soup is offered.	- A jacket potato with baked beans (384 kcal) for a jacket potato with baked beans and cheese (633 kcal).	80%
Soup & Sandwiches (n=15)		- A smoked ham & mustard sandwich (262 Kcal) for a BLT (355 kcal).	80%
Sweet snacks (n=18)	- Swaps offered are as close in nature to the originally selected item as possible.<br>- Swaps offered are at least 50kcal less than the originally selected item.	- A strawberry yoghurt (141 kcal) for a granola and yoghurt pot (241 kcal).	72%
Savoury snacks (n=20)		- A packet of Propercorn Sea Salted (87 kcal) for a packet of Walkers Ready Salted (171 kcal)	75%
Drinks (n=18)	- Swaps offered for drinks will almost always be the lower-energy version of the originally selected drink.<br>- Where diet equivalents are unavailable or do not meet the criteria, swaps judged to be relatively close in flavour to the original item will be offered.<br>- Swaps offered are at least 50kcal less than the originally selected item.	- Coke Zero (<1 kcal) for Classic Coca-Cola (210 kcal).<br>- Fanta Zero (<1 kcal) for San Pellegrino orange flavour (129 kcal).	50%

6.3 Intervention

Participants will be randomly allocated to receive one of the following messages when swaps were offered (Figure 1):

- Control: No swaps offered
- Swaps: "How about a swap?"
- Swaps + PACE: "*How about a swap? Save [x] calories = [y] min walk*".

The price of each item will be presented beneath the image of the item. This was based on the price list provided to us by a real canteen based in the UK. Energy information will be presented next to price for all items, to all groups.

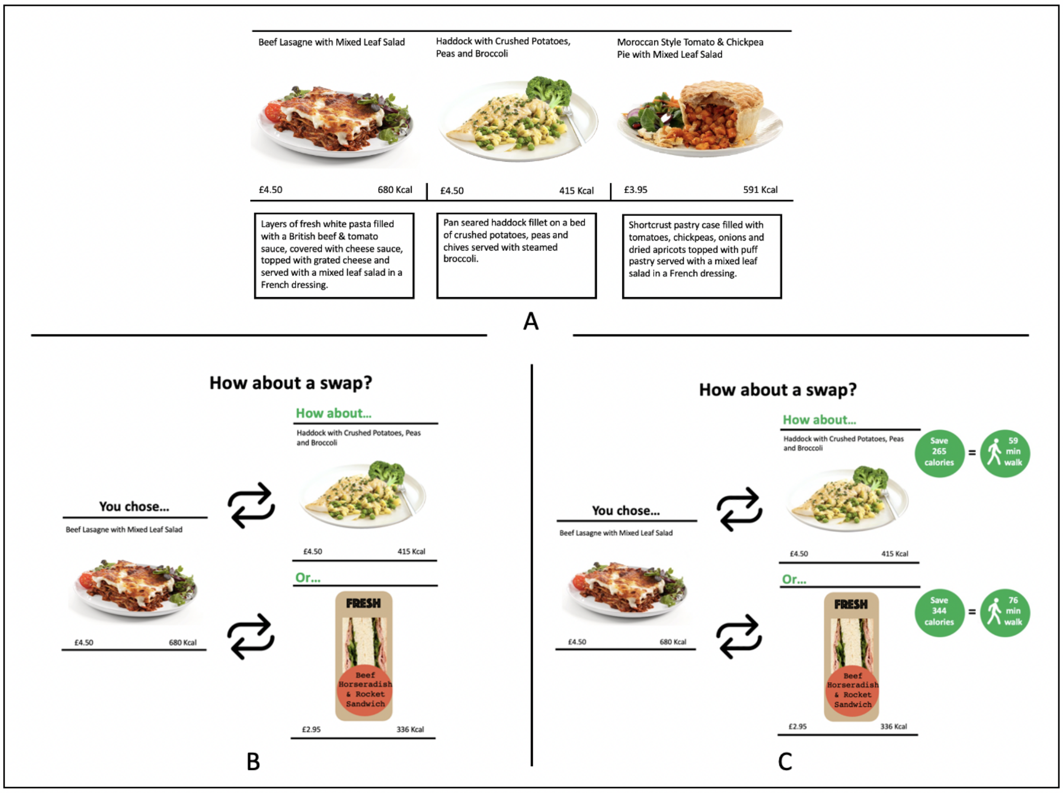

Figure 2: An example of the intervention when selecting a main hot meal. (A) Main hot meal menu day 1; (B) the swaps condition and; (C) the swaps+PACE condition.

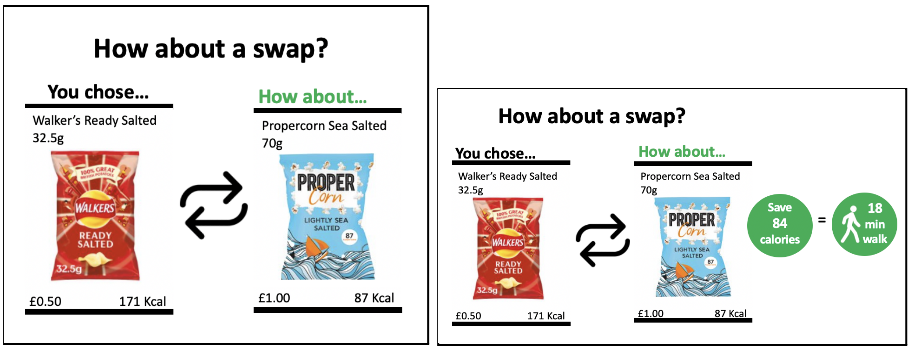

(a) (b)

Figure 3: An example of the intervention when selecting a savoury snack (Walker's Ready Salted) in which 1 swap is offered, by condition. (a) the Swaps condition; (b) the Swaps + PACE condition.

The PACE message will indicate minutes of moderate walking. The PACE information is calculated using the METs Calculator (https://metscalculator.com) developed by the Compendium of Physical Activity (Ainsworth et al., 2011). The input for the calculator is based on the average UK adult, who according to the Office for National Statistics (ONS) is 39 years old and weighs 77kg (ONS, 2010). The description of the activity selected is "2.8-3.2mph moderate pace, firm surface". Using these figures, moderate walking burns approximately 4.5 calories per minute.

6.4 Pre-task questions & exit survey

Before making their selections participants' subjective in-the-moment feelings of hunger will be assessed using a visual analog scale (VAS), a graphic rating scale first developed by Hayes and Patterson in 1921. A VAS usually comprises a series of horizontal lines with words anchored at each end, "not at all" on the far left and "extremely" on the far right. Participants then use this scale to respond to questions related to their subjective feelings of hunger, pain etc. In this study, subjective ratings of appetite will be collected using 1 item "How hungry do you feel?" (adapted from Stubbs et al., 2000). Participants will be asked to make a mark along a virtual line using a slider to reflect the in-the-moment intensity of their feelings (Appendix A). Higher scores indicating higher-levels of hunger. This method has been used in previous studies to assess in-the-moment appetite (Purcell et al., 2014).

The exit survey will include (i) an attention check; (ii) questions on the acceptability of the intervention; (iii) a demographic characteristics questionnaire (Appendix C).

Attention check

The first question on the exit survey will be an attention check. Participants in the two intervention groups will be asked to indicate whether or not they were offered a swap.

Acceptability

Acceptability will be assessed by asking participants the extent to which they agree it would be acceptable for their employer to (a) implement a pre-ordering system for their workplace canteen and (b) offer them swaps for their food choices. Only those in the intervention groups will be asked question (b). Response options will be on a scale from 1 (completely unacceptable) to 5 (completely acceptable).

Demographic information

Participants will then answer questions relating to their characteristics on the following measures (i) dietary restraint (ii) physical activity (iii) Body Mass Index (BMI).

(i) A shortened version The Dietary Intent Scale (DIS) will be used to assess dietary restraint. The DIS is a nine-item measure created by Stice (1998) based on the Dutch Restrained Eating Scale (DRES; Van Strien, Frijters, Van Staveren, Defares, & Deurenberg, 1986). The scale is internally (α = 0.94) and temporally reliable (1-month test-retest = 0.92). Due to time constraints, the scale was reduced to 3 items. The scale was shortened by assessing the correlation between items, removing highly correlated items and then checking Cronbach's alpha. The Cronbach's alpha remained high after retaining 3 key items (highlighted below) at α = 0.81. Response options will be: 1= Never, 2= Seldom, 3= Sometimes, 4= Often, 5= Always. Higher scores indicate more dietary restraint (score range = 3-15).

<u>The Dietary Intent Scale</u>

Please choose the response that best describes your eating behaviour in the past 6 months:

1. I take small helpings in an effort to control my weight
2. I hold back at meals in an attempt to prevent weight gain
3. **I limit the amount of food I eat in an effort to control my weight (retained)**
4. I sometimes avoid eating in an attempt to control my weight
5. I skip meals in an effort to control my weight
6. I sometimes eat only one or two meals a day to try to limit my weight
7. I eat diet foods in an effort to control my weight
8. **I count calories to try to prevent weight gain (retained)**
9. **I eat low-calorie foods in an effort to avoid weight gain (retained)**

(ii) Physical activity will be assessed using the Scottish Physical Activity Screening Questionnaire (Scot-PASQ) (NHS, 2013). The tool was developed and validated by NHS Health Scotland in partnership with The University of Edinburgh. The tool has up to 2 questions depending on answers provided. The first question asks: “How many days in the past week have you been physically active for a total of 30 minutes or more?” (see Appendix C for full question wording). Participants responding four days or less are asked the following second question: “Have you been physically active for at least two and a half hours (150 minutes) over the course of the past week?” Yes/No. Those answering 5, 6 or 7 days to question 1 or those answering ‘yes’ to question 2 will be categorised as “meeting guidelines”.

(iii) Body Mass Index (BMI): Participants will be asked to indicate their height (in centimeters or feet and inches) and their weight (in kilograms or stones and pounds). BMI will be calculated dividing the weight in kg by the square of height in meters.

Finally participants will provide demographic information including: age, sex, ethnicity, and highest level of education completed (see Appendix C).

7. STATISTICS AND ANALYSIS

7.1. Sample Size

A power calculation was conducted for the primary outcome; the difference in energy (kcal) ordered between each of the trial arms (Table 4). The power calculations account for the BH correction. As it is not possible to apply the Benjamini-Hochberg (BH) correction before having the actual data, the following steps were taken. First, an uncorrected (p<0.05) calculation was conducted, which provided an upper bound on power. Then, a calculation using the Bonferroni correction (p<0.017), which provides a lower bound. Power using the BH correction will lie somewhere between these two bounds. In this study, the maximum number of comparisons made will be 3.

The minimum detectable effect size (MDES) was initially set at a 35-40kcal reduction. While a 50kcal reduction would be a clinically relevant calorie reduction for adults (Hill et al., 2003), the MDES was reduced to 35-40kcal because the relative effect between the experimental arms was expected to be smaller than the effect between the experimental arms and control. Reducing the MDES to 35-40kcal will allow for the detection of this reduction if present. Baseline (mean 423kcal & SD; 236kcal) energy estimates were taken from a Pilot RCT conducted in 6 workplace canteens across the UK (Vasiljevic et al., 2018).

Based on the input outlined above, the required total sample size was 2,214 to detect a ~35 kcal at an alpha level of 0.05 (an uncorrected analysis) or a 40kcal difference at an alpha level of 0.017 (Bonferroni) change in energy ordered. A final sample of n= 2,453 was recruited to account for 10% attrition (2,214 + 221). Sample size calculations were completed using STATA version 16.

Table 3: Sample size estimates for the primary outcome, energy ordered, based on a range of energy-reductions with upper, middle lower bounds for required n.

**Av Energy Reduction**	**N per arm**	**Total n**	**In Budget**	**N per arm**	**Total n**	**In Budget**	**N per arm**	**Total n**	**In Budget**
	**Upper Bound (P<0.05)**			**Middle (P<0.033)**			**Lower Bound (P<0.016)**		
30kcal	973	**2919**	No	1,096	**3,288**	No	1505	**4515**	No

31kcal	911	**2733**	No	1,027	3,081	No	1227	**3681**	No
32kcal	855	**2565**	No	964	2,892	No	1151	**3453**	No
33kcal	804	**2412**	No	906	2,718	No	1083	**3294**	No
34kcal	758	**2274**	No	854	2,562	No	1020	**3060**	No
35kcal	715	**2145**	Yes	806	2,418	No	963	**2889**	No
36kcal	676	**2028**	Yes	762	2,286	No	910	**2730**	No
37kcal	640	**1920**	Yes	721	2,163	Yes	862	**2586**	No
38kcal	607	**1821**	Yes	684	2,052	Yes	817	**2451**	No
39kcal	576	**1728**	Yes	649	1,947	Yes	776	**2328**	No
40kcal	548	**1644**	Yes	617	1,851	Yes	738	**2214**	Yes

7.2. Outcome Measures

Primary outcome

The primary outcome was the total energy (kcal) of items ordered by each of the three groups.

Secondary outcomes

(a) Secondary outcome (a) was the number of swaps accepted (restricted to the groups being offered swaps), controlling for the number of swaps offered.
(b) Secondary outcome (b) was the proportion of participants ordering a lunch that meets the Public Health England (PHE, 2018) guideline of containing 600 kcal or less in each of the three groups.
(c) Secondary outcome (c) was Likert scale ratings of intervention acceptability by intervention groups

Exploratory outcomes

(a) Exploratory outcome (a) was an interaction analysis examining differences in intervention effects (for the primary outcome) by each of the following variables: sex, age, ethnicity, education, BMI, physical activity level, dietary restraint, and hunger.

(b) Exploratory outcome (b) was the effect of price difference between the initially selected item and the swap offered by intervention (restricted only to groups being offered swaps) on swap acceptance.
(c) Exploratory outcome (c) was the acceptance of swaps offered (restricted to the groups being offered swaps), separately for each of the 6 categories: (i) main meals; (ii) jacket potatoes; (iii) soup & sandwiches; (iv) sweet snacks; (v) savoury snacks and; (vi) drinks."

7.3 Statistical Analysis

A pre-specified statistical plan will be registered in advance of the analysis on AsPredicted.org. Analysis will be carried out using STATA version 16 or SPSS (version 25). All participants will be asked to select their hypothetical lunch for the day. A response will be deemed incomplete if the participant fails to complete an order and 'checkout'. Participants with incomplete responses will be excluded from the analysis. Estimates of comparative effectiveness will be reported as either mean differences, regression coefficients or odds ratios (OR) with 95% confidence intervals (CI). The attention check question will be analysed descriptively.

Primary outcome

Primary outcome (a) will be analysed using ANOVA. Post-hoc tests will be run. Although post-hoc adjustments are not required when making pre-defined comparisons (Li et al., 2017), the Benjamini-Hochberg step-up procedure will be applied to correct for multiple testing. This procedure is described in section 7.4.

Secondary outcomes

Secondary outcome (a) will be analysed using ordinal logistic regression given that the number of swaps offered is 1-6 and accepted ranges from 0-6. Only those offered a swap will be included in this analysis. The number of swaps offered will be controlled for. If the data does not meet the assumption of parallel trends required to use ordinal logistic regression, a poisson regression (or negative binomial if there is an abundance of zero value) for count data will be used.

Secondary outcome (b) will be analysed using logistic regression, two regressions will be run, changing the reference group so that we can examine differences between the intervention groups as well as comparing each of these to the control.

Secondary outcome (c) will be analysed using ordinal logistic regression, again two regressions will be run, changing the reference group so that we can examine differences between the intervention groups as well as comparing each of these to the control.

Exploratory outcomes

Exploratory outcome (a) will be analysed using two-way ANOVA with post-hoc tests to test for interaction effects between total energy ordered (the primary outcome) and the following demographic variables: sex (male vs female), ethnicity (white vs non-white), income (below £25K vs above £25K per year), education (none/secondary vs higher), age (above vs below the median), dietary restraint (above vs below the median), physical activity level (meeting vs not meeting guidelines), and BMI (BMI of >=30kg/m2 vs BMI<30). Separate models will be conducted for each of the 8 demographic variables.

Exploratory outcome (b) will be analysed using multilevel logistic regression. Only those offered a swap will be included in this analysis. Given that participants can select more than one item and swap price varies (i.e. can be cheaper or more expensive than the original item), data will be reshaped so the unit of analysis becomes 'swap'. We will calculate the difference in price between each initially selected item and the swap offered (in pounds and pence). Negative values will reflect a cheaper swap offered and positive values a more expensive swap offered. For main hot meals where two swaps are offered, price difference will be calculated using the price of the swap that is accepted. Where no swap is accepted, the average price difference for the two different swaps offered will be used. Conducting analysis at the swap-level means that there may be multiple observations per participant in the data. To account for this, we use the cluster option to indicate that observations are clustered by participant and that observations may be correlated within participant but would be independent between participants. As such, we will cluster using participant ID. Before running the regression model, we will check the distribution of price difference by condition to ensure that the distributions match one another. A logistic regression will then be conducted to test whether price difference predicts swap acceptance for those offered a swap in the swaps and swaps+PACE groups. An interaction term for intervention and price difference will also be included. Robust standard errors will be used to account for potential heterogeneous effects.

Exploratory outcome (c) will be analysed using logistic regression. A separate model will be run for each of the six menus. The reference category will be the control group in all models.

7.4 Correction for multiple comparisons: The Hochberg procedure

A Benjamini-Hochberg (BH) correction (Hochberg, 1995) will be used to adjust the $p<0.05$ significant level for multiple comparisons in the ANCOVA and regression models. The Hochberg procedure has been shown to adequately control the alpha level when making multiple comparisons for tests with positively correlated dependent variables (FDA, 2017). The Hochberg procedure works as follows: It takes the *p*-values from each comparison and arranges them in ascending order. Rather than comparing these against a fixed significance

threshold (usually p<0.05), the Hochberg procedure compares them with a linearly increasing vector from 0.05/k (where k is the number of comparisons) to 0.05. Thus when making 3 comparisons (m=3), this first p-value must be <0.017, the next p-value must be <0.033 and the third p-value must be <0.05 to be declared significant.

Example: in the current 3-arm RCT (1 control group, 2 treatment groups) with 1 primary outcome we will conduct 3 statistical tests, as we compare each of the 2 treatment coefficients against the control coefficient and against one another. Suppose the 3 statistical tests produce *p*-values of: **H1: 0.06; H2: 0.015; H3: 0.005.**

These values are ranked in increasing order, and compared. The below table describes this process.

#	p-value	Original significance threshold	New significance threshold	p-value < new significance threshold?
1	0.005	0.05	0.05*(1/3) = 0.017	Yes
2	0.015	0.05	0.05*(2/3) = 0.033	Yes
3	0.06	0.05	0.05*(3/3) = 0.05	No

The lowest p-value (0.005) must be < 0.017 to be accepted (which it **is**)
The 2nd lowest must be < 0.033 to be accepted (which it **is**)
The 3rd lowest must be < 0.05 to be accepted (which it **isn't**) – everything thereafter would also be rejected.

8. DATA MANAGEMENT

8.1. Access to Data

Direct access will be granted to authorised representatives from the University College London for monitoring and/or audit of the study to ensure compliance with regulations.

8.2. Data Handling and Record Keeping

All study data will be captured in a password protected secure database. The participants will be identified by a unique study specific number and/or code in any database. Personal data will not be collected in this study. Research data and records will be retained for at least three years after publication or public release of the work of the research and reviewed thereafter.

9. QUALITY CONTROL AND QUALITY ASSURANCE PROCEDURES

The study will be conducted in accordance with the current approved protocol, relevant regulations and standard operating procedures.

10. ETHICAL AND REGULATORY CONSIDERATIONS

10.1. Approvals

Approval has been granted by the UCL Ethics Committee (Reference number:12861/005).

10.2. Participant Confidentiality

We will not hold personal data (including IP address), but Prolific Academic does (e.g. full name, email address). When individuals register with Prolific Academic, they agree to their privacy policy. When participants consent to take part in this study, they will **not** be asked for their name, email address or any other identifiable information. They will only be asked for their Prolific ID so that we can ensure that they will be paid for their participation. Although researchers at UCL will not be able to identify any individuals in this study, we are technically collecting pseudo-anonymised data. This is because our data (containing Prolific ID) could theoretically be linked with the personal data Prolific Academic holds about participants via the Prolific ID. Any data linking is highly unlikely as we will collect all of our data through REDCap which will not be accessible by anyone other than the research team. Equally, Prolific Academic does not share personal information about their panel members with researchers. We will take precautions to ensure that no-one is identifiable from our data. For example, once data collection has ceased, we will remove the Prolific ID from the spreadsheet to minimise any risk.

All documents will be stored securely (UCL's Data Safe-Haven) requiring password-protected access and will be only accessible by study staff and authorised personnel.

10.3. Expenses and Benefits

In line with Prolific Academic's guidelines, participants will be reimbursed £0.50 for their time. All incentives are awarded only once the study has been completed.

11. FINANCE AND INSURANCE

11.1. Funding

This study is funded by the annual research grant provided by the Behavioural Insights Team.

11.2. Insurance

This is a simple online task and we cannot foresee any unintended or adverse effects due to participation. University College London maintains Public Liability and Professional Liability insurance which will operate in this respect.

12. PUBLICATION POLICY

Key findings from this study will go into a PhD thesis, which will be held in UCL's publicly accessible library. We will also aim to publish this paper in an open access journal.

13. REFERENCES

Appendices

Appendix A: Screening Survey & VAS Hunger Scale

1. Do you have any dietary restrictions? Including: Vegetaria, Pescetarian, Vegan, Dairy/lactose free, Sugar-free, Gluten-free, Eggs allergy, Nut allergy, other food allergies, other dietary restrictions.
    - Yes/ No
2. Are you in full or part-time employment?
    - Yes/No
3. How hungry do you feel?
    - [slider] 0 "Not at all" on the far left, and 100 "Extremely" on the far right.

Appendix B: Task instructions

Welcome

We'd like you to imagine that you arrive at work on Monday morning to receive this email…

Hi Team,

We've set-up a new online ordering system for the canteen. Please login to your account and choose your lunch for today. The canteen will have your order ready for collection.

Thank you,
The Canteen Manager

- We'd like you to select your lunch for the day.

- You do not need to order from every menu.
- Please order what you would typically have for lunch during a single work day.
- To order from a menu please click “begin survey” beside the menu you wish to order from.
- When you have finished your order, please click “begin survey” beside “Checkout”.

[Next Page]

Appendix C: Exit Survey & debriefing text

1. [Attention check; swaps & swaps+PACE conditions only] Were you offered a swap? Yes/No
2. [Acceptability; All participants] “How acceptable do you think it would it be for your employer to implement a pre-ordering system for your workplace canteen like the one you have just used? (Response options: 1 = completely unacceptable, 2 = unacceptable, 3= neither acceptable nor unacceptable, 4= acceptable, 5= completely acceptable).
3. [Acceptability; swaps & swaps+PACE conditions] “How acceptable do you think it would be for your employer to offer you swaps for your food choices in your workplace canteen?” (Response options: 1 = completely unacceptable, 2 = unacceptable, 3= neither acceptable nor unacceptable, 4= acceptable, 5= completely acceptable).
4. [Dietary Restraint; All participants] “Please choose the response that best describes your eating behaviour in the past 6 months”:
    1. I limit the amount of food I eat in an effort to control my weight (limit)
    2. I count calories to try to prevent weight gain (count)
    3. I eat low-calorie foods in an effort to avoid weight gain (calorie)

    (Response options: 1 = Never , 2 = Seldom , 3= Sometimes, 4= Often , 5= Always)
5. [Physical Activity Level SCOT-PASQ q1; All participants] How many days in the past week have you been physically active for a total of 30 minutes or more? Remember vigorous activity (e.g. running, jogging, vigorous swimming, cycling) counts for double. (Response: 0-7 days)
6. [Physical Activity Level SCOT-PASQ q2; if q1 4 days or less] Have you been physically active for at least two and a half hours (150 minutes) over the course of the past week?

    This can be achieved in a number of ways, such as:
    - 30 minutes moderate physical activity (e.g. fast walking, easy cycling, easy swimming) on most days of the week
    - A two and a half hour walk or cycle at the weekend
    - A combination of activity options equalling a minimum of 150 minutes.

    Remember vigorous activity (e.g. running, jogging, vigorous swimming, cycling) counts for double. (Response: Yes/No)
7. [BMI; All participants] What is your height? (cms; feet & inches), Prefer not to say

8. [BMI; All participants] What is your weight? (kg; stones & pounds), Prefer not to say
9. [Sex; All participants] I am…
   - Male
   - Female
   - Other
   - Prefer not to say
10. [Age; All participants] What age are you? (text box), Prefer not to say
11. [Ethnicity; All participants] Which best describes your ethnic group or background?
    - White
    - Mixed
    - Asian
    - Black
    - Other
    - Prefer not to say
12. [Education; All participants] Please indicate the highest level of education you have completed to date.
    - No formal qualifications
    - Secondary school/GCSE
    - College/ A levels
    - Undergraduate degree (BA/BSc/other)
    - Graduate degree (MA/MSc/MPhil/other)
    - Doctorate degree (PhD/MD/other)
    - Prefer not to say

Debriefing text

Thank you for your Participation!

The aim of this study was to investigate the effect of offering participants lower-calorie swaps for their lunchtime selections on a pre-ordering website, on the total calories ordered for their lunch.

In this study, participants were randomly assigned to one of the following groups: Group 1 were offered lower-calorie swaps for their selections if a suitable alternative was available; Group 2 were also offered lower-calorie swaps and these swaps were accompanied by a physical activity equivalent message indicating the number of minutes walking that could be saved by accepting the swap offered; Group 3: were not offered any swaps and ordered their lunch as usual.

If you have any questions about this research, please email sarah.breathnach.17@ucl.ac.uk.

Please click "submit" to return to your Prolific Academic dashboard.

Appendix D: Full menus

MAIN HOT MEALS

Day 1								
**Swap 1**								
**Name**	**Price**	**Kcal**		**Swap Offered**	**Price**	**Kcal**	**Kcal saved**	**PACE**
Beef Lasagne & mixed leaf salad, french dressing	£4.50	680		Haddock with crushed potatoes, peas & broccoli	£4.50	415	265	59
Haddock with crushed potatoes, peas & broccoli	£4.50	415		Tuna & sweetcorn sandwich	£2.45	308	107	24
Moroccan style tomato & chickpea pie, mixed leaf salad, french dressing	£3.95	591		Haddock with crushed potatoes, peas & broccoli	£4.50	415	176	40
**Swap 2**								
**Name**	**Price**	**Kcal**		**Swap Offered**	**Price**	**Kcal**	**Kcal saved**	**PACE**
Beef Lasagne & mixed leaf salad, french dressing	£4.50	680		Beef, horseradish & rocket sandwich	£2.95	336	344	76
Haddock with crushed potatoes, peas & broccoli	£4.50	415		Jacket potato with (plain) tuna	£1.78	344	71	16
Moroccan style tomato & chickpea pie, mixed leaf salad, french dressing	£3.95	591		Falafel wrap	£2.95	355	236	52
**Day 2**								
**Swap 1**								
**Name**	**Price**	**Kcal**		**Swap Offered**	**Price**	**Kcal**	**Kcal saved**	**PACE**
Chicken Katsu curry, basmati rice	£4.50	673		Shepards pie with garden peas	£4.50	536	137	30
Shepards pie with garden peas	£4.50	536		Tomato and basil soup with bread (petit pain)	£1.35	235	301	67
Vegetable stir-fry, prawn crackers	£3.95	590		Shepards pie with garden peas	£4.50	536	54	12
**Swap 2**								
**Name**	**Price**	**Kcal**		**Swap Offered**	**Price**	**Kcal**	**Kcal saved**	**PACE**
Chicken Katsu curry, basmati rice	£4.50	673		Coronation chicken sandwich	£2.95	421	252	56
Shepards pie with garden peas	£4.50	536		Smoked ham & mustard sandwich	£2.45	262	274	61
Vegetable stir-fry, prawn crackers	£3.95	590		Tomato and basil soup with bread (petit pain)	£1.35	235	355	79
**Day 3**								
**Swap 1**								
**Name**	**Price**	**Kcal**		**Swap Offered**	**Price**	**Kcal**	**Kcal saved**	**PACE**
Beef carvery, roasties, mash potatoes, carrots, cabbage, broccoli, Yorkshire pudding,				Sticky chicken in a khobez wrap with quinoa tabbouleh and feta	£4.50	451	380	84

horseradish & gravy	£4.50	831		cheese.				
Sticky chicken in a Khobez wrap with quinoa tabbouleh and feta cheese	£4.50	451		Roast Chicken Salad Sandwich	£2.75	302	149	33
Puttanesca linguini with ciabatta	£3.95	682		Sticky chicken in a khobez wrap with quinoa tabbouleh and feta cheese.	£4.50	451	231	51
**Swap 2**								
**Name**	**Price**	**Kcal**		**Swap Offered**	**Price**	**Kcal**	**Kcal saved**	**PACE**
Beef carvery, roasties, mash potatoes, carrots, cabbage, broccoli, Yorkshire pudding, horseradish & gravy	£4.50	831		Beef, horseradish & rocket sandwich	£2.95	336	495	110
Sticky chicken in a Khobez wrap with quinoa tabbouleh and feta cheese	£4.50	451		Tomato and basil soup with bread (petit pain)	£1.35	235	216	48
Puttanesca linguini with ciabatta	£3.95	682		Tomato and basil soup with bread (petit pain)	£1.35	235	447	99
**Day 4**								
**Swap 1**								
**Name**	**Price**	**Kcal**		**Swap Offered**	**Price**	**Kcal**	**Kcal saved**	**PACE**
Bangers, mash, rich onion gravy	£4.50	692		Aubergine chickpea penne	£3.95	419	273	61
Chicken Gyros served with bulgar wheat salad	£4.50	626		Aubergine chickpea penne	£3.95	419	207	46
Aubergine chickpea penne	£3.95	419		Falafel wrap	£2.95	355	64	14
**Swap 2**								
**Name**	**Price**	**Kcal**		**Swap Offered**	**Price**	**Kcal**	**Kcal saved**	**PACE**
Bangers, mash, rich onion gravy	£4.50	692		Jacket potato with pulled pork	£1.75	505	187	41
Chicken Gyros served with bulgar wheat salad	£4.50	626		Roast chicken salad sandwich	£2.75	302	324	72
Aubergine chickpea penne	£3.95	419		Tomato and basil soup with bread (petit pain)	£1.35	235	184	41
**Day 5**								
**Swap 1**								
**Name**	**Price**	**Kcal**		**Swap Offered**	**Price**	**Kcal**	**Kcal saved**	**PACE**
Chinese chicken curry, white rice	£4.50	681		Tomato, goats cheese & basil tart, leaf salad	£3.95	461	220	49
Fisherman’s pie, potato crust served with steamed	£4.50	601		Tomato, goats cheese & basil tart, leaf salad	£3.95		140	31

broccoli						461		
Tomato, goats cheese & basil tart, leaf salad	£3.95	461		Cheddar ploughman's sandwich	£2.75	405	56	12
**Swap 2**								
**Name**	**Price**	**Kcal**		**Swap Offered**	**Price**	**Kcal**	**Kcal saved**	**PACE**
Chinese chicken curry, white rice	£4.50	681		Coronation chicken	£2.95	421	260	58
Fisherman's pie, potato crust served with steamed broccoli	£4.50	601		Jacket Potato with tuna	£1.78	344	257	57
Tomato, goats cheese & basil tart, leaf salad	£3.95	461		Tomato and basil soup with bread (petit pain)	£1.35	235	226	50

**SOUP & SANDWICH MENU**									
**Name**	**Price**	**Kcal**		**Swap Offered**	**Price**	**Kcal**	**Price diff**	**Kcal saved**	**PACE**
Bacon Lettuce Tomato (BLT)	£2.75	355		Smoked Ham & Mustard	£2.45	262	£0.30	93	20
Beef, Horseradish & rocket	£2.95	336		Smoked Ham & Mustard	£2.45	262	£0.50	74	16
Cheddar Ploughman's	£2.75	405		Smoked Ham & Mustard	£2.45	262	£0.30	143	32
Chicken & Stuffing	£2.45	335		Smoked Ham & Mustard	£2.45	262	£0.00	73	16
Chicken, Bacon & Stuffing	£2.95	422		Chicken & Stuffing	£2.45	335	£0.50	87	19
Coronation Chicken	£2.95	421		Roast Chicken Salad	£2.75	302	£0.20	119	26
Egg & Cress	£2.45	322		Smoked Ham & Mustard	£2.45	262	£0.00	60	13
Falafel Wrap (red pepper houmous)	£2.95	355		Smoked Ham & Mustard	£2.45	262	£0.50	93	20
Ham, Cheddar & Pickle	£2.75	398		Smoked Ham & Mustard	£2.45	262	£0.30	136	30
Prawn Mayo	£3.20	345		Smoked Ham & Mustard	£2.45	262	£0.75	83	18
Roast Chicken Salad	£2.75	302		Tomato and Basil soup with bread	£1.35	235	£1.40	67	15
Smoked Ham & Mustard	£2.45	262		No swap					
Southern Fried Chicken Wrap	£3.20	449		Roast Chicken Salad	£2.75	302	£0.45	147	32
Tomato & Basil soup with bread	£1.35	235		No Swap					

Tuna & Sweetcorn	£2.45	308		Tomato and Basil soup with bread	£1.35	235	£1.10	73	16

JACKET POTATO MENU									
**Name**	**Price**	**Kcal**		**Swap Offered**	**Price**	**Kcal**	**Price diff**	**Kcal saved**	**PACE**
Plain Jacket Potato	£0.88	275		No Swap					
Jacket Potato with butter/spread	£0.98	319		No Swap					
Jacket Potato with cheese	£1.78	524		Jacket Potato with baked beans	£1.30	384	£0.48	140	31
Jacket Potato with baked beans	£1.30	384		Jacket Potato with butter/spread	£0.98	319	£0.32	65	14
Jacket Potato with baked beans & cheese	£2.20	633		Jacket Potato with baked beans	£1.30	384	£0.90	249	55
Jacket Potato with tuna mayo	£1.78	479		Jacket Potato with (plain) tuna	£1.78	344	£0.00	135	30
Jacket Potato with tuna mayo & cheese	£2.68	728		Jacket Potato with tuna mayo	£1.78	479	£0.90	249	55
Jacket Potato with (plain) tuna	£1.78	344		Plain Jacket Potato	£0.88	275	£0.90	69	15
Jacket Potato with (plain) tuna & cheese	£2.68	593		Jacket Potato with (plain) tuna	£1.78	344	£0.90	249	55
Jacket Potato with pulled pork	£1.75	505		Jacket Potato with baked beans	£1.30	384	£0.45	121	27

SWEET SNACK MENU									
**Name**	**Price**	**Kcal**		**Swap Offered**	**Price**	**Kcal**	**Price diff**	**Kcal saved**	**PACE**
Banana	£0.60	105		No Swap					
Blueberry Crumble Muffin	£1.90	471		Oat, Raisin & Sicilian Lemon Cookie	£1.65	309	£0.25	162	36
Broderick's Caramental	£1.20	237		Mamma Loretti's Chocolate (15g)	£1.00	79	£0.20	158	35
Broderick's Chocolatey Solid Brick	£1.20	217		Mamma Loretti's Chocolate (15g)	£1.00	79	£0.20	138	30
Broderick's Peanut	£1.20	261		Mamma Loretti's	£1.00	78	£0.20	183	40

Chunk				Hazelnut (15g)					
Chocolate Brownie	£1.70	283		Broderick's Chocolatey Solid Brick	£1.20	217	£0.50	66	15
Chocolate Chunk Cookie	£1.35	373		Oat, Raisin & Sicilian Lemon Cookie	£1.65	309	-£0.30	64	14
Flapjack	£1.95	295		Granola (Fruit & Yoghurt)	£1.25	214	£0.70	81	18
Fruit Salad (brakes)	£1.00	74		No Swap					
Granola (Fruit & Yoghurt)	£1.25	214		Ubley Yoghurt Strawberry	£0.65	141	£0.60	73	16
Mamma Loretti's Chocolate (15g)	£1.00	79		No Swap					
Mamma Loretti's Hazelnut (15g)	£1.00	78		No Swap					
Mamma Loretti's Tiramisu (15g)	£1.00	80		No Swap					
Oat, Raisin & Sicilian Lemon Cookie	£1.65	309		Granola (Fruit & Yoghurt)	£1.25	214	£0.40	95	21
Raspberry & White Chocolate Muffin	£1.75	369		Oat, Raisin & Sicilian Lemon Cookie	£1.65	309	£0.10	60	13
Ubley Yoghurt Peach	£0.65	141		Fruit Salad (brakes)	£1.00	74	-£0.35	67	15
Ubley Yoghurt Raspberry	£0.65	141		Fruit Salad (brakes)	£1.00	74	-£0.35	67	15
Ubley Yoghurt Strawberry	£0.65	141		Fruit Salad (brakes)	£1.00	74	-£0.35	67	15

SAVOURY SNACK MENU									
**Name**	**Price**	**Kcal**		**Swap Offered**	**Price**	**Kcal**	**Price diff**	**Kcal saved**	**PACE**
Eat Real Hummus Chips Chili Cheese 45g	£1.10	213		Walkers Cheese & Onion (32.5g)	£0.50	161	£0.60	52	11
Eat Real Hummus Chips Chilli & Lemon 45g	£1.10	202		Propercorn Sun Dry Tomato & Chilli (20g)	£1.00	88	£0.10	114	25
Eat Real Hummus Chips Sea Salt 45g	£1.10	217		Propercorn Sea Salted 20g	£1.00	87	£0.10	130	29
Eat Real Lentil Chips Creamy Dill 40g	£1.10	189		Propercorn Sour Cream & Black Pepper 20g	£1.00	96	£0.10	93	20
Eat Real Lentil Chips Tomato & Basil 40g	£1.10	186		Propercorn Sun Dry Tomato & Chilli (20g)	£1.00	88	£0.10	98	22

Eat Real Lentil Mango & Mint 40g	£1.10	178		Propercorn Sea Salted (20g)	£1.00	87	£0.10	91	20
Eat Real Quinoa Chips Sour Cream & Chive 30g	£1.10	165		Propercorn Sour Cream & Black Pepper 20g	£1.00	96	£0.10	69	15
McCoys Flame Grilled Steak 47.5g	£0.80	252		Popchips BBQ 23g	£1.32	97	-£0.52	155	34
McCoys Flame Thai Sweet Chicken 47.5g	£0.80	261		Eat Real Lentil Mango & Mint (40g)	£1.10	178	-£0.30	83	18
Pipers Anglesey Sea Salt 40g	£0.85	213		Propercorn Sea Salted 20g	£1.00	87	-£0.15	126	28
Pipers Burrow Hill Cider Vinegar & Sea Salt 40g	£0.85	209		Propercorn Sea Salted 20g	£1.00	87	-£0.15	122	27
Pipers Lye Cross Cheddar & Onion 40g	£0.85	213		Walkers Cheese & Onion 32.5g	£0.50	161	£0.35	52	11
Popchips BBQ 23g	£1.32	97		No Swap					
Popchips Sour Cream & Onions 23g	£1.32	95		No Swap					
Propercorn Sea Salted 20g	£1.00	87		No Swap					
Propercorn Sour Cream & Black Pepper 20g	£1.00	96		No Swap					
Propercorn Sun Dry Tomato & Chilli 20g	£1.00	88		No Swap					
Walkers Cheese & Onion 32.5g	£0.50	161		Propercorn Sour Cream & Black Pepper 20g	£1.00	96	-£0.50	65	14
Walkers Ready Salted 32.5g	£0.50	171		Propercorn Sea Salted 20g	£1.00	87	-£0.50	84	18
Walkers Salt & Vinegar 32.5g	£0.50	169		Propercorn Sea Salted 20g	£1.00	87	-£0.50	82	18

DRINKS MENU									
**Name**	**Price**	**Kcal**		**Swap Offered**	**Price**	**Kcal**	**Price diff**	**Kcal saved**	**PACE**
Cawston Press Ginger Beer	£1.30	89		Sprite Free	£0.80	3	£0.50	86	19
Cawston Press Sparkling Rhubarb	£1.30	86		Oasis Zero	£1.20	15	£0.10	71	16
Classic Coke	£1.25	210		Coke zero	£0.80	2	£0.45	208	46

Coke Zero	£1.10	2		No swap					
Diet coke	£0.80	0		No swap					
Dr Pepper Zero	£0.80	1		No swap					
Fanta Zero	£0.80	10		No swap					
Innocent Juice - Orange smooth	£1.55	121		Fanta Zero	£0.80	10	£0.75	111	24
Innocent Juice - Orange with bits	£1.55	123		Fanta Zero	£0.80	10	£0.75	113	25
Life sparkling	£0.65	0		No swap					
Life still	£0.65	0		No swap					
Oasis Summer fruits	£1.20	85		Oasis Zero	£1.20	15	£0.00	70	15
Oasis Summer fruits Zero	£1.20	15		No swap					
Redbull Zero	£1.70	10		No swap					
Redbull	£1.80	110		RedBull Zero	£1.70	10	£0.10	100	22
San Pellegrino - Arancia Rossa	£1.30	73		Fanta Zero	£0.80	10	£0.50	63	14
San Pellegrino - Limonata	£1.30	71		Sprite Free	£0.80	3	£0.50	68	15
Sprite Free	£0.80	3		No swap					
