# Supplementary material for: Strategies to reduce the energy content of foods pre-ordered for lunch in the workplace: a randomised controlled trial in an experimental online canteen
Source: Int J Behav Nutr Phys Act. 2022 May 12;19:54. doi: 10.1186/s12966-022-01257-5 (PMC9096740; doi:10.1186/s12966-022-01257-5)
Supplement: Supplementary file 3 — Additional file 3. [file 12966_2022_1257_MOESM3_ESM.docx]

**Additional File 3: Supplementary Figures & Tables**

**Strategies to reduce the energy content of foods pre-ordered for lunch in the workplace: a randomised controlled trial in an experimental online canteen**

**Breathnach S, Lally P, Llewellyn C.H., Sutherland A, & Koutoukidis D.A.**

**Attention checks**

Table 1: First Attention check. Number (%) of participants responding correctly and incorrectly to the question: “Were you offered a swap?”

| **Condition** | **Correct** |
| --- | --- |
| Swaps (n=711) | 710 (99%) |
| Swaps+PACE (n=713) | 709 (99%) |

Table 2: Number of participants ordering from each menu and average energy ordered by group (n=2,150).

|  | **Mains** | | | **Jackets** | **Sandwiches** | **Sweet** | **Savoury** | **Drinks** |
| --- | --- | --- | --- | --- | --- | --- | --- | --- |
|  | *mean kcal ± SD (n) | | | | | | | |
| Control  (726) | 631 ± 109 (253) | | | 572 ± 122  (195) | 354 ± 60  (381) | 241 ± 117 (435) | 177 ± 53 (319) | 53 ± 69 (626) |
| Swaps  (713) | 573 ± 153 (277) | | | 545 ± 124  (192) | 354 ± 63 (345) | 216 ± 119 (403) | 169 ± 59 (282) | 44 ± 63 (611) |
| Swaps +PACE  (711) | 563 ± 153 (295) | | | 521 ± 126  (197) | 343 ± 63  (323) | 209 ± 120 (397) | 161 ± 58 (291) | 36 ± 56 (627) |
| **Total** | 587 ± 141 (825) | | | 546 ± 126 (584) | 351 ± 62 (1,049) | 223 ± 119 (1,235) | 169 ± 57 (892) | 44 ± 63 (1,867) |

SD = Standard Deviation , *means unadjusted for kcal of first item ordered

**Exploratory outcome (a) mediation analyses**

Table 3: Exploratory outcome (a) Sex. Interaction effect of condition by **sex** (n=2,145).

|  | **df** | **F** | **partial η^2^** | **P-value** |
| --- | --- | --- | --- | --- |
| Condition | 2 | 10.28 | 0.010 | >0.001* |
| Sex (female) | 1 | 3.04 | 0.001 | 0.081 |
| Sex*Condition | 2 | 0.895 | 0.001 | 0.409 |
| Error | 2,138 | - | - | - |

Note: * Benjamini-Hochberg-adjusted significance level.

Table 4: Exploratory outcome (a) age. Interaction effect of condition by **age** (n=2,149).

|  | **df** | **F** | **partial η^2^** | **P-value** |
| --- | --- | --- | --- | --- |
| Condition | 2 | 10.41 | 0.010 | >0.001* |
| Age (above median) | 1 | 81.22 | 0.037 | >0.001* |
| Age*Condition | 2 | 0.306 | 0.000 | 0.736 |
| Error | 2,142 | - | - | - |

Note: * Benjamini-Hochberg-adjusted significance level.

Table 5: Exploratory outcome (a) ethnicity. Interaction effect of condition by **ethnicity** (n=2,139).

|  | **df** | **F** | **partial η^2^** | **P-value** |
| --- | --- | --- | --- | --- |
| Condition | 2 | 6.24 | 0.006 | 0.002* |
| Ethnicity (White) | 1 | 16.28 | 0.008 | >0.001* |
| Ethnicity*Condition | 2 | 0.300 | 0.000 | 0.741 |
| Error | 2,132 | - | - | - |

Note: * Benjamini-Hochberg-adjusted significance level.

Table 6: Exploratory outcome (a) education. Interaction effect of condition by **education** (n=2,142).

|  | **df** | **F** | **partial η^2^** | **P-value** |
| --- | --- | --- | --- | --- |
| Condition | 2 | 11.36 | 0.011 | >0.001* |
| Education (Undergrad +) | 1 | 0.429 | 0.000 | 0.513 |
| Education*Condition | 2 | 0.007 | 0.000 | 0.993 |
| Error | 2,135 | - | - | - |

Note: * Benjamini-Hochberg-adjusted significance level.

Table 7: Exploratory outcome (a) BMI. Interaction effect of condition by **BMI** (n=2,135).

|  | **df** | **F** | **partial η^2^** | **P-value** |
| --- | --- | --- | --- | --- |
| Condition | 2 | 8.68 | 0.008 | <0.001* |
| BMI (>=BMI 30kg/m2) | 1 | 0.337 | 0.000 | 0.561 |
| BMI*Condition | 2 | 0.279 | 0.000 | 0.756 |
| Error | 2,128 | - | - | - |

Note: * Benjamini-Hochberg-adjusted significance level.

Table 8: Exploratory outcome (a) physical activity. Interaction effect of condition by **physical activity (PA)** (n=2,149)**.**

|  | **df** | **F** | **partial η^2^** | **P-value** |
| --- | --- | --- | --- | --- |
| Condition | 2 | 11.91 | 0.011 | >0.001* |
| Physical activity (meeting PA guidelines) | 1 | 3.842 | 0.002 | 0.050 |
| Physical activity *Condition | 2 | 1.192 | 0.001 | 0.304 |
| Error | 2,142 | - | - | - |

Note: * Benjamini-Hochberg-adjusted significance level.

Table 9: Exploratory outcome (a) Hunger scale rating (0-100) . Interaction effect of condition by **hunger rating** (n=2,150).

|  | **df** | **F** | **partial η^2^** | **P-value** |
| --- | --- | --- | --- | --- |
| Condition | 2 | 10.52 | 0.011 | >0.001* |
| Hunger rating (above median) | 1 | 9.842 | 0.002 | 0.002* |
| Hunger rating*Condition | 2 | 1.027 | 0.001 | 0.358 |
| Error | 2,143 | - | - | - |

Note: * Benjamini-Hochberg-adjusted significance level.

Table 10: Exploratory outcome (a) dietary restraint score (0-15). Interaction effect of condition by **dietary restraint score** (n=2,150).

|  | **df** | **F** | **partial η^2^** | **P-value** |
| --- | --- | --- | --- | --- |
| Condition | 2 | 10.70 | 0.010 | >0.001* |
| Dietary restraint score (above median) | 1 | 36.815 | 0.017 | >0.001* |
| Dietary restraint*Condition | 2 | 2.57 | 0.002 | 0.077 |
| Error | 2,143 | - | - | - |

Note: * Benjamini-Hochberg-adjusted significance level.

**Exploratory outcome (c) Swap acceptance by menu**

Table 11: Exploratory outcome (c). Swap acceptance by condition for the **main menu** (n=572).

|  | **Odds Ratio** | **95% Confidence Interval** | **P-value** |
| --- | --- | --- | --- |
| Condition (PACE) | 0.97 | 0.64 to 1.48 | 0.886 |
| Constant | 0.23 | 0.17 to 0.31 | >0.001* |

Note: * Benjamini-Hochberg-adjusted significance level.

Table 12: Exploratory outcome (c). Swap acceptance by condition for **jacket potato menu** (n=368).

|  | **Odds Ratio** | **95% Confidence Interval** | **P-value** |
| --- | --- | --- | --- |
| Condition (PACE) | 2.77 | 1.40 to 5.47 | 0.003* |
| Constant | 0.08 | 0.04 to 0.13 | >0.001* |

Note: * Benjamini-Hochberg-adjusted significance level.

Table 13: Exploratory outcome (c). Swap acceptance by condition for **sandwiches menu** (n=563).

|  | **Odds Ratio** | **95% Confidence Interval** | **P-value** |
| --- | --- | --- | --- |
| Condition (PACE) | 2.13 | 1.15 to 3.94 | 0.016* |
| Constant | 0.06 | 0.04 to 0.10 | >0.001* |

Note: * Benjamini-Hochberg-adjusted significance level.

Table 14: Exploratory outcome (c). Swap acceptance by condition for **sweet snack menu** (n=537).

|  | **Odds Ratio** | **95% Confidence Interval** | **P-value** |
| --- | --- | --- | --- |
| Condition (PACE) | 1.66 | 1.07 to 2.58 | 0.023 |
| Constant | 0.18 | 0.13 to 0.25 | >0.001* |

Note: * Benjamini-Hochberg-adjusted significance level.

Table 15: Exploratory outcome (c). Swap acceptance by condition for **savoury snack menu** (n=434).

|  | **Odds Ratio** | **95% Confidence Interval** | **P-value** |
| --- | --- | --- | --- |
| Condition (PACE) | 1.04 | 0.58 to 1.86 | 0.887 |
| Constant | 0.13 | 0.09 to 0.20 | >0.001* |

Note: * Benjamini-Hochberg-adjusted significance level.

Table 16: Exploratory outcome (c). Swap acceptance by condition for **drinks menu** (n=462).

|  | **Odds Ratio** | **95% Confidence Interval** | **P-value** |
| --- | --- | --- | --- |
| Condition (PACE) | 3.17 | 1.73 to 5.81 | 0.000* |
| Constant | 0.07 | 0.04 to 0.12 | >0.001* |

Note: * Benjamini-Hochberg-adjusted significance level.

**Sensitivity analysis**

Table 17a: Pre-specified primary analysis: ANOVA testing differences in energy ordered between groups. Means, Standard deviations and 95% confidence intervals for energy ordered (n=2150).

| Group | *Mean (SD) Final Kcals* | *Group* | *Mean (SD) Final Kcals* | *Mean Difference*  *(95% CI)* | | *P-Value* |
| --- | --- | --- | --- | --- | --- | --- |
| Control | 828 (332) | Swaps | 767 (299) | -61 (-21 to -100) | | 0.001* |
| Control | 828 (332) | Swaps+PACE | 748 (306) | -80 (-40 to -120) | | <0.001* |
| Swaps | 767 (299) | Swaps+PACE | 748 (306) | -20 (20 to -59) | | 0.558 |

Note: * Benjamini-Hochberg-adjusted significance level.
